# Towards accurate indel calling for oncopanel sequencing through an international pipeline competition at precisionFDA

**DOI:** 10.1038/s41598-024-58573-y

**Published:** 2024-04-08

**Authors:** Binsheng Gong, Samir Lababidi, Rebecca Kusko, Khaled Bouri, Sarah Prezek, Vishal Thovarai, Anish Prasanna, Ezekiel J. Maier, Mahdi Golkaram, Xingqiang Sun, Konstantinos Kyriakidis, João Paulo Kitajima, Sayed Mohammad Ebrahim Sahraeian, Yunfei Guo, Elaine Johanson, Wendell Jones, Weida Tong, Joshua Xu

**Affiliations:** 1 Division of Bioinformatics and Biostatistics, Office of Research, National Center for Toxicological Research, Office of the Chief Scientist, Office of the Commissioner, U.S. Food and Drug Administration, Jefferson, AR 72079 USA; 2 Health Informatics Staff, Office of Data, Analytics, and Research, Office of Digital Transformation, Office of the Commissioner, U.S. Food and Drug Administration, Silver Spring, MD 20993 USA; 3 Cellino Biotech, 750 Main Street, Cambridge, MA 02143 USA; 4 Office of Regulatory Science and Innovation, Office of the Chief Scientist, Office of the Commissioner, U.S. Food and Drug Administration, Silver Spring, MD 20993 USA; 5 Booz Allen Hamilton, McLean, VA 22102 USA; 6 Illumina, Inc., San Diego, CA 92122 USA; 7 Genetalks, Changsha, Hunan China; 8 UC Santa Cruz Genomics Institute, Santa Cruz, CA 95060 USA; 9 Mendelics, Av. Braz Leme, 1631-Santana, São Paulo, SP 02511-000 Brazil; 10 Roche Sequencing Solutions, Santa Clara, CA 95050 USA; 11 Q squared Solutions Genomics, 2400 Elis Road, Durham, NC 27703 USA

**Keywords:** Indel calling pipeline, Next-generation sequencing, Precision medicine, Personalized medicine, precisionFDA challenge, Bioinformatics, Personalized medicine, Data processing, Quality control, High-throughput screening

## Abstract

Accurately calling indels with next-generation sequencing (NGS) data is critical for clinical application. The precisionFDA team collaborated with the U.S. Food and Drug Administration’s (FDA’s) National Center for Toxicological Research (NCTR) and successfully completed the NCTR Indel Calling from Oncopanel Sequencing Data Challenge, to evaluate the performance of indel calling pipelines. Top performers were selected based on precision, recall, and F1-score. The performance of many other pipelines was close to the top performers, which produced a top cluster of performers. The performance was significantly higher in high confidence regions and coding regions, and significantly lower in low complexity regions. Oncopanel capture and other issues may have occurred that affected the recall rate. Indels with higher variant allele frequency (VAF) may generally be called with higher confidence. Many of the indel calling pipelines had good performance. Some of them performed generally well across all three oncopanels, while others were better for a specific oncopanel. The performance of indel calling can further be improved by restricting the calls within high confidence intervals (HCIs) and coding regions, and by excluding low complexity regions (LCR) regions. Certain VAF cut-offs could be applied according to the applications.

## Introduction

An indel is a type of genetic mutation that occurs when nucleotides are added or removed from a deoxyribonucleic acid (DNA) sequence. Indels within the coding regions can lead to changes in protein structure and function^1^. Indels in non-coding regions can also alter the structure and function of regulatory elements, such as promoters, enhancers and microribonucleic acid (RNA) targeting sites, which are involved in the gene expression regulation.

When it occurs in cancer-associated genes, indels can disrupt the normal function and/or regulation of these genes, leading to the activation of oncogenic pathways and the suppression of tumor suppressor pathways^2,3^. The study of indels in cancer can provide insight into the underlying genetic changes that drive cancers and identification of specific indels can help with the diagnosis, prognosis, and treatment of cancers^4,5^.

The presence of specific indels can be used to distinguish between different types of cancer and help to guide treatment decisions; can indicate a poor prognosis; and can provide valuable information for precision medicine in cancer by providing insight into the underlying genetic changes^6,7^ for developing targeted cancer therapies. Inaccurate indel calls can lead to misleading or false conclusions about the functional consequences of a given genetic variant and can have important implications for the clinical interpretation of genetic test results.

Unfortunately, accurately calling indels with next-generation sequencing (NGS) data can be challenging due to the complex nature of indels, along with short read length, alignment errors, technical variability, and low variant allele frequency (VAF)^8,9^. In addition to the improvements that can be made in the sequencing technologies, such as longer reads, higher coverage, and polymerase chain reaction (PCR)-free library preparation, the development of variant calling algorithms and bioinformatics pipelines remains critical to accurately identify true indels (recall) and the ability to correctly identify indels without generating false positive results (precision).

In some applications, a high recall is more important, such as in a medical diagnosis, where a false negative call could result in missing the opportunity of a specific treatment^6,10^. In other applications, high precision is more appreciated, where higher false positives called will lead to over estimation of tumor mutational burden (TMB)^11,12^. Undoubtedly, researchers are trying their best to make both the recall and precision as high as possible, but according to different applications, the indel calling pipelines may be tuned toward either way or optimized for a balance between precision and recall.

There are several strategies that can be used to improve the performance of indel calling with NGS data^8,13^. One approach is to use more sensitive variant-calling algorithms that are specifically designed to handle indels. These algorithms often use specialized techniques, such as realignment or local assembly to better handle the variable length and complexity of indels. Another strategy is to use multiple independent methods for variant calling, and to carefully evaluate the results using appropriate quality metrics and filtering criteria. This can help to reduce the false positive rate (FPR) and false negative calls and improve the overall accuracy of the indel calls.

A third strategy is to compare the results of technical replicates of the same samples that were sequenced multiple times and/or with different laboratory technologies. By measuring the consistency of indel calling, researchers can reduce the number of false positive calls. Comparing results from multiple bioinformatics pipelines against the indel benchmarking known sets can be very helpful to better understand the relationship between the indel calling performance and indel properties, such as size, location, and frequency. It can also help to identify any potential issues or sources of errors that may cause false calls, such as PCR amplification, capture, fragmentation, contamination, and loss of material.

The high value of clinically actionable information obtained by oncopanel sequencing makes it a crucial tool for precision oncology^7,14^. With the surge in availability of oncopanels, it is critical to ensure that they have been thoroughly tested and are properly used. The U.S. Food and Drug Administration (FDA) has initiated the Sequencing Quality Control phase II (SEQC2) project^15^ to develop standard analysis protocols and quality control metrics for fit-for-purpose use of NGS data, including oncopanel sequencing to inform regulatory science research and precision medicine.

The Oncopanel Sequencing Working Group (OSWG) of the FDA-led SEQC2 consortium has developed a reference sample suitable for benchmarking oncopanels and comprehensively assessed the analytical performance of several oncopanels^16^. Given that indels have not been studied as much as single nucleotide variants (SNVs), it is important that the tools for indel-calling be rigorously evaluated and optimized. To this end, precisionFDA and the FDA’s National Center for Toxicological Research (NCTR) have successfully completed the NCTR Indel Calling from Oncopanel Sequencing Data Challenge^17,18^. This challenge asked the participants to develop, validate, and benchmark indel calling pipelines to identify indels in the oncopanel sequencing datasets. The NCTR team used the indels that have been reported in the SEQC2^16^ study, as well as an extended indel set, which was manually reviewed by a group of researchers in an internal project, to evaluate the indel calling performance of the precisionFDA challenge submissions. There are several ways to evaluate the performance of indel calling with NGS data for the precisionFDA challenge. One approach is to use a known set of indels developed by the SEQC2-OSWG to assess the precision, recall, and overall performance of the indel calls of each submission. It may also be useful to compare the results of different indel callers or to use multiple independent methods for indel calling and evaluate the agreement between them.

## Results

### Study design

**Figure 1:**
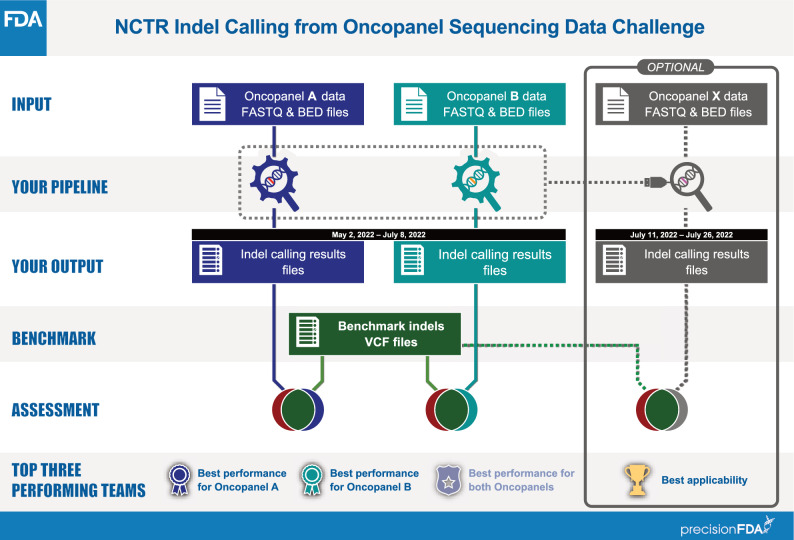
Study design.

The “NCTR Indel Calling from Oncopanel Sequencing Data Challenge” was announced on the precisionFDA website and multiple social media platforms publicly. A precisionFDA contributor account was required to participate in the challenge. Twenty-one participants (teams or individuals) signed up for the challenge. The challenge had two phases. In phase 1, participants were provided with two sets of oncopanel sequencing data and were asked to develop indel calling pipelines that are optimized for either or both datasets (Oncopanel A and Oncopanel B, Fig. 1). Each set corresponds to a unique oncopanel. In phase 2, participants were given two weeks to run their pipelines with the frozen-in parameters on a third oncopanel sequencing dataset (Oncopanel X) if their pipelines were developed (and results were submitted) for both Oncopanel A and B.

### Top performers

**Figure 2:**
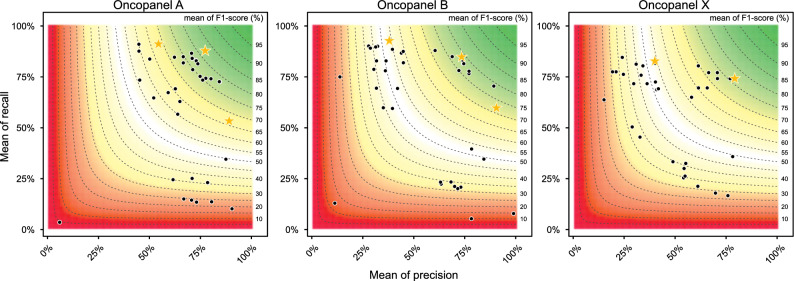
Performance of all valid submissions. X-axis is the mean precision and y-axis is the mean recall. The contour map shows the mean F1-score where the color gradient from green to red is proportionate to the mean F1-score from high to low. The top performers are marked with golden stars.

The performance was measured with recall, precision, and F1-score (see “Methods”), using a known set of indels (see “Methods”). The performance of all valid submissions is shown in Fig. 2. The top performers are marked as golden stars. Some submissions were very close to the top performers in terms of precision, recall and F1-score, respectively. Overall, submissions for Panel A had high precision. All three panels had some submissions with low recall rates. One submission for Panel B had precision of 99%, but the recall was less than 8%.

**Figure 3:**
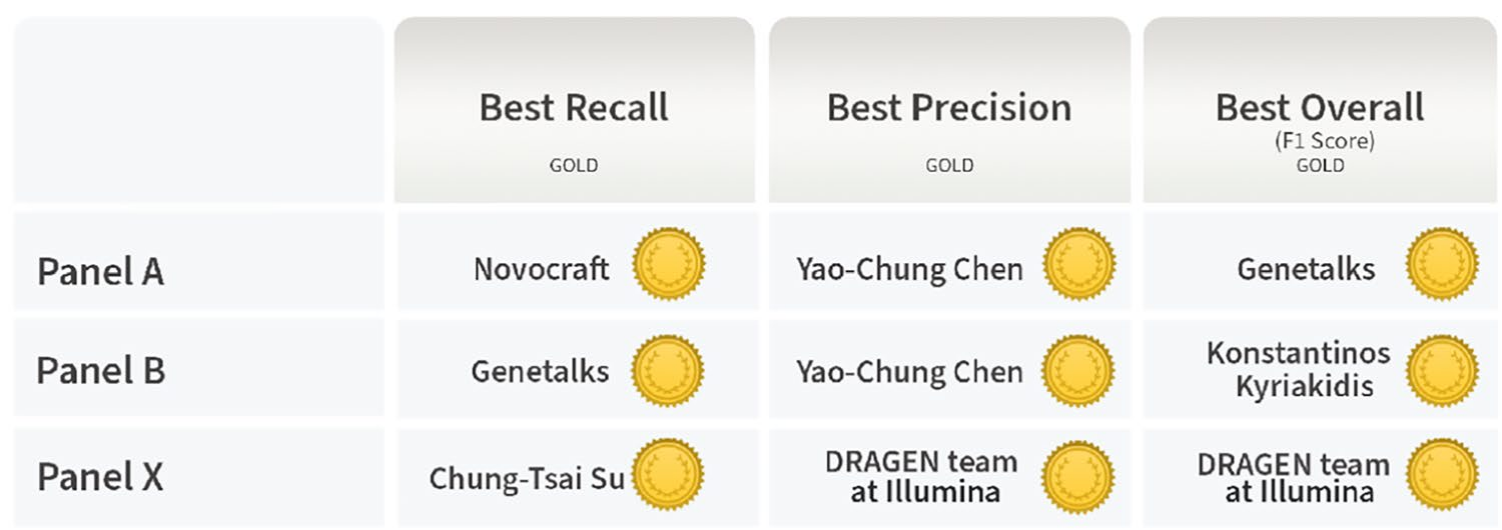
Image on the result page of the precisionFDA challenge’s website. Top performers for Best Recall, Best Precision, and Best Overall for Panel A, Panel B, and Panel X.

The top performers, including top recall, top precision, and top overall performance, for each panel were selected within the submissions whose average F1-score is above median, accordingly. As shown in Fig. 3^18^, a total of six top performers were selected, three for Panel A, three for Panel B, and two for Panel X. Three pipelines were top performers across the panel or metrics.

### Performance evaluation by statistical modeling

**Figure 4:**
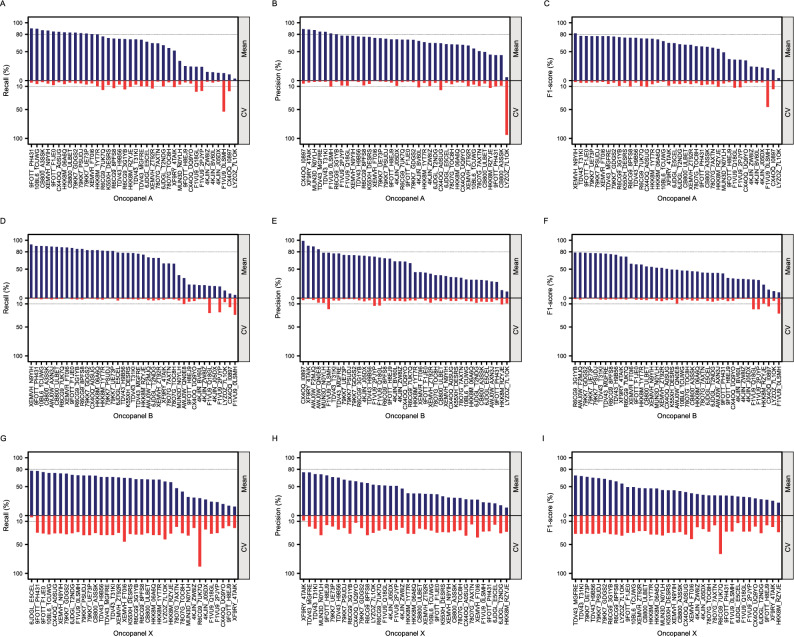
The mean and CV of the three measurements for Oncopanel A, B, and X. The blue bars represent the mean of the measurements, and the red bars represent the CV of the measurements. The pipelines (x-axis) were sorted by the mean of the measurements for each oncopanels accordingly. Two reference lines are shown as 80% for mean and 10% for CV.

In this study, we observed that pipeline performance can vary not only between teams but also within teams, particularly with different laboratories and with different libraries within laboratories (Fig. 4).

Although the top performers were chosen based on the highest numerical mean score across laboratories and libraries in each category, there were several pipelines whose mean scores were not substantially different from top performers. We used a statistical model (see “Methods”) to test whether certain pipelines exhibit similar performance to the top performer for each oncopanel according to each performance metric. In the model, each pipeline (shown in the column “Pipeline2” in Supplementary Table 1–9) was compared with the top performer (shown as the “Pipeline1”) for each oncopanel by each of three performance metrics. We found that there were several pipelines that had no statistically significant difference in their mean performance with top performers, except for Oncopanel B when the metric Precision was applied. For instance, the difference in mean recall between the top performer and 6 pipelines in Oncopanel A were not statistically significant, meaning that the difference in performance among these pipelines was likely due to random chance (Fig. 4A, Supplementary Table 1). Similar observations were also seen for Oncopanel A by Precision, Oncopanel A by F1-score, Oncopanel B by Recall and Oncopanel B by F1-score (Fig. 4B–F, Supplementary Tables 2–6).

The different laboratories and the libraries within laboratories in which the pipelines from the participants were run presented variability in their performance metrics. For most of the pipelines in Oncopanels A and B, we see that the coefficient of variation (CV) is around 10%, which is a reasonable threshold for variability, except for the team F1VU9, which exhibited a relatively larger CV than other teams (Fig. 4A–F). However, unsurprisingly Oncopanel X exhibited much larger variability than Oncopanels A and B for most of the pipelines (Fig. 4G–I, Supplementary Table 7–9). This is due to the frozen-in parameters requirement, where participants had much less information about the Oncopanel X than in the case of Oncopanels A and B. We noticed here that the larger CV seen in the performance metrics for Oncopanel X had the implication of causing a rather large number of pipelines, with mean performance to be not statistically significantly different from the top performer (Fig. 4G, Supplementary Table 7). The mean and CV values for Fig. 4 can be found in Supplementary Table 10, where the top performers were highlighted.

### Factors which impact the indel calling performance

#### Performance was higher in the High Confidence Interval (HCI) and coding regions

**Figure 5:**
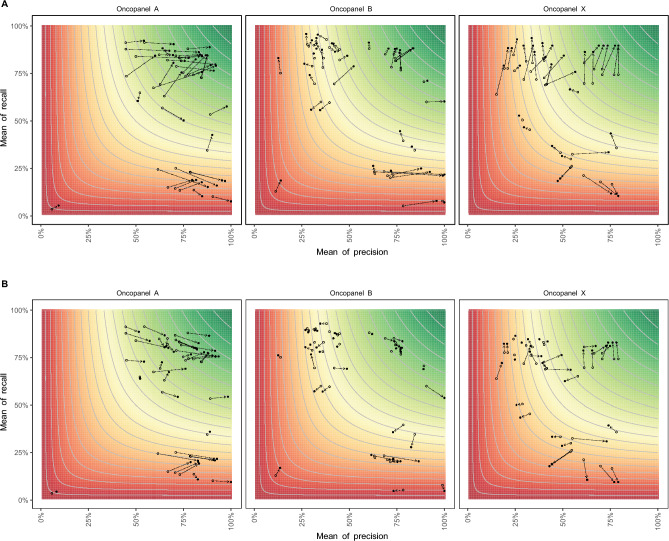
Performance comparison among different genomic regions. The x-axis is the mean of precision for each pipeline, and the y-axis is the mean of recall for each pipeline. The open dots represent the measurements of evaluation regions, while the solid dots represent the measurements of (**A**) HCI regions, or (**B**) coding regions. The contour lines and color represent the mean of F1-score for each pipeline, where the color gradient from green to red is proportionate to the mean F1-score from high to low.

We examined the performance of indels within the Genome in a Bottle (GIAB) HCI and coding regions (according to University of California, Santa Cruz (UCSC) Genome Browser). As expected, the precision, recall, and the F1-score were significantly higher in both HCI and coding regions (Fig. 5). As shown in Table 1, by restricting indel calling in the HCI regions, the recall increased by 2.03 ± 5.81%, 3.40 ± 3.61%, 7.20 ± 7.59%, the precision increased by 11.75 ± 7.14%, 3.66 ± 9.27%, 2.63 ± 5.89%, and F1-score increased 5.41 ± 6.57%, 1.87 ± 3.64%, 2.87 ± 6.13%, for Oncopanel A, B, X, respectively. The performance also increased by restricting variant calling in coding regions, especially the precision increased 9.28 ± 6.01% and F1-score increased 3.50 ± 3.72% for Oncopanel A, mainly because Oncopanel A is a large panel containing a larger portion of non-coding regions, while the other two Oncopanels are more focused on the coding regions by design.

**Table 1:** Performance increases by restricting variant calling in HCI regions and coding regions.

Oncopanel	Recall (%)		Precision (%)		F1-score (%)	
	Average	Maximum	Average	Maximum	Average	Maximum
Performance increases by restricting variant calling in HCI regions						
Oncopanel A	2.03 ± 5.81	12.42	11.75 ± 7.14	27.50	5.41 ± 6.57	17.41
Oncopanel B	3.40 ± 3.61	9.57	3.66 ± 9.27	36.04	1.87 ± 3.64	10.31
Oncopanel X	7.20 ± 7.59	17.81	2.63 ± 5.89	18.82	2.87 ± 6.13	13.44
Performance increases by restricting variant calling in coding regions						
Oncopanel A	− 0.60 ± 3.34	5.73	9.28 ± 6.01	21.68	3.50 ± 3.72	10.13
Oncopanel B	− 0.77 ± 2.41	7.07	0.91 ± 4.72	18.22	0.73 ± 2.48	4.72
Oncopanel X	1.58 ± 5.32	9.29	0.55 ± 5.85	17.81	0.21 ± 5.42	9.19

#### Indel calling was difficult in the low complexity regions (LCRs)

**Figure 6:**
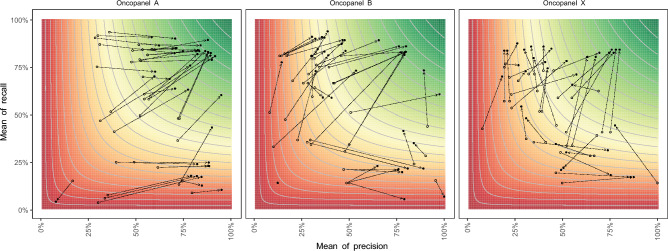
Performance comparison between low complexity coding regions (coding LCRs) and evaluation regions that exclude the coding LCRs. The x-axis is the mean of precision for each pipeline, and the y-axis is the mean of recall for each pipeline. The contour lines represent the mean of F1-score for each pipeline, where the color gradient from green to red is proportionate to the mean F1-score from high to low.

LCRs have repetitive sequences, which can cause difficulties in accurately aligning the reads to the reference genome, leading to false positive and false negative indel calls. LCRs also pose challenges for indel calling because they are often prone to systematic errors in sequencing, such as PCR-induced duplications or deletions. This can lead to incorrect annotation of the size and location of the indels, as well as overestimation of the frequency of indels. As shown in Fig. 6, it is not surprising that the performance of indel calling was significantly lower in the LCR compared with non-LCR.

#### “False positives” were not reproducible

In this study, any indel call that was not in the known indel set was a “false positive”. These assignments were supported by their irreproducibility and lack of annotation. We assessed reproducibility among library replicates and pipelines with precision above 50%. Indels reported by pipelines with precision below 50% were excluded from our analysis due to the heightened likelihood of such results containing a greater proportion of uniquely identified false positive calls. A vast majority (87%) of the false positives were called less than 5% of the time among all library replicate—pipeline combinations (408 for Oncopanel A, 264 for Oncopanel B, and 153 for Oncopanel X, Supplementary Fig. 1). This assignment was also supported by the low annotation rate with Human Genome Variation Society (HGVS) database using SnpEff (see “Methods”), where only about 5% of the false positives being annotated. In comparison, 100% of the benchmarking known indels were successfully annotated.

#### “False negatives” may be caused by issues with panel capture

A small number of “false negatives” were not identified by any of the pipelines, despite being included in the benchmarking known set. Upon visual inspection using Integrative Genomics Viewer (IGV) (an example is shown in Supplementary Fig. 2), we discovered that there were few reads covering the positions of these “false negatives” at the ends of these reads. However, in the cell-line data used to identify the benchmarking known set, there were a sufficient number of reads covering the positions, with enough reads indicating the presence of the indels. Therefore, the “false negatives” may have been caused by factors such as panel capture or other issues, rather than the pipelines themselves.

#### Recall may be marginally better on higher VAF

It is widely accepted that indels with higher VAF can be more accurately detected than those with lower VAF. To determine the effect of VAF on recall, we applied a series of VAF cut-offs to the benchmarking known indels. As shown in Supplementary Fig. 3, we observed a slight increase in recall for Oncopanel A and almost no change for Oncopanel B. However, an unexpected decrease in recall for Oncopanel X was observed, which was due to the limited number of indels that remained when the VAF cut-off was set at 10% or higher. Some of these indels were located in challenging regions, such as non-HCI, non-coding, and LCR regions.

## Discussion

Double-blinded performance evaluation was used in this challenge, where participants were asked to develop bioinformatics pipelines for indel calling and submit results to the precisionFDA team. The results were then evaluated by evaluation team at NCTR, who was blinded to the identity of the participants, and the results are used to rank the participants and determine the top performers. Double-blinded performance evaluation provided a fair and objective assessment of the participants' performance, since it eliminated the potential for bias by the assessors or by the participants. It also helped ensure that the results were based on the performance, rather than the reputation of the participants or the institutions they represent.

A total of 1000 + indels were taken from an extended benchmarking known set of indels and the published known indel set for Sample A^16^, where 267 indels (Supplementary Table 13) were located in the targeted regions of the three oncopanels used in the challenge. If sequencing the reference Sample A with a larger panel, more known indels from this extensive indel “truth” set can be utilized to provide a comprehensive and robust evaluation of the panel and the performance of indel calling pipelines, allowing for improved accuracy and sensitivity.

The benchmarking known set of indels is composed of two parts, one of which (about half) was taken from our previous publication^16^ and the other was collected through a recent manual review effort. Although the participants were not informed of the published portion of the benchmarking set, researchers may still be able to optimize their pipelines using the published indels. The unreleased portion of the benchmarking set helped to maintain the independence of the evaluation, allowing for a more objective evaluation of performance. This "half-and-half" benchmarking set of indels enables researchers to improve their pipelines by identifying false positive or false negative calls, and to objectively evaluate performance with an independent set of known indels.

Indel calling can perform differently on different genome regions for a number of reasons. One reason is that the accuracy of indel calls can be affected by the sequencing depth and the signal-to-noise ratio of the data. Regions with low coverage or low signal-to-noise ratio may be more difficult to confidently call indels, leading to a higher rate of false negatives or false positives. Another reason is that some genome regions may be more prone to indels due to the presence of repetitive or structural variation. For example, indels are more common in repetitive regions, such as satellite DNA, and may be more difficult to accurately align and call due to the presence of multiple copies of the same sequence. Additionally, some indel callers may be more sensitive to indels in certain types of genome regions, such as exons or conserved non-coding regions, compared to others. This can also affect indel calling performance on different genome regions. Finally, indel calling performance may also be affected by the presence of other types of genomic variations, such as single nucleotide variations or structural variations, which can interfere with the accuracy of the indel calls.

Variant Call Format (VCF) is widely used in the research community due to its well-defined structure. However, VCF files produced by different pipelines or software may have different formatting, making it difficult to directly compare them, even by using tools designed specifically for comparing VCF files, such as rtg-tools^19^ and bcftools^20^. Despite providing guidance and a template to participants, submissions still varied in formatting. These formatting differences could lead to discrepancies in the representation of the same indels in different submissions, which may could result in an inaccurate evaluation of results.

Researchers may use the detailed information about the known indels (Supplementary Table 13) to explore additional analysis for improving their own indel calling pipelines. However, when the targeted genomic regions are smaller, fewer known indels can be utilized to carry out the stratified performance analysis by the indel type, length, or VAF distribution. In such scenarios, we strongly suggest that researchers refrain from drawing conclusions based on a small number of indels.

## Conclusions

The performance of indel calling has improved significantly with advancements in NGS technology and bioinformatics algorithms. Some indel calling pipelines performed well on all three oncopanels, while others were better for a specific oncopanel. Although we were set to pick one top performer for each category, the performance of many other pipelines was close to the top performers, which makes them a top cluster of performers. The performance of indel calling can be improved by restricting the calls within HCI and coding regions, and by excluding LCR regions. Generally, indels with higher VAF may be called with higher confidence, and certain VAF cut-off should be applied according to the applications.

## Methods

### The aim, design, and setting of the study

Participants were provided with two sets of oncopanel sequencing data and were asked to develop indel calling pipelines, which are optimized for either or both datasets (Oncopanel A and Oncopanel B). Each set is generated by a unique oncopanel different from the other set. Participants had the option to validate their pipelines with the frozen-in parameters on a third oncopanel sequencing dataset (Oncopanel X) in addition to the first two datasets. All participants needed to have a contributor account on precisionFDA to download the data and submit the results.

### The characteristics of participants

The challenge went live on May 2, 2022. Phase 1 ran for two months from May 2 to July 8, 2022 and Phase 2 ran two weeks from July 11 to July 26, 2022. Participants were asked to test and fine tune up to three pipelines per team on the Phase 1 data, and directly apply the fixed pipelines to Phase 2 data within a limited time. In total, submissions of 48 pipelines from 21 teams were received. However, two teams misunderstood the challenge and submitted results with complex formatting non-adherence, and one other team's submission is on hg38. Thus 7 submissions were removed from the evaluation. Table 2 shows the number of valid submissions. The submissions were de-identified and passed to the evaluation team.

**Table 2:** Number of valid submissions.

	Oncopanel A	Oncopanel B	Oncopanel X
Number of teams	17	18	17
Number of pipelines	38	41	36

### Description of data files

**Figure 7:**
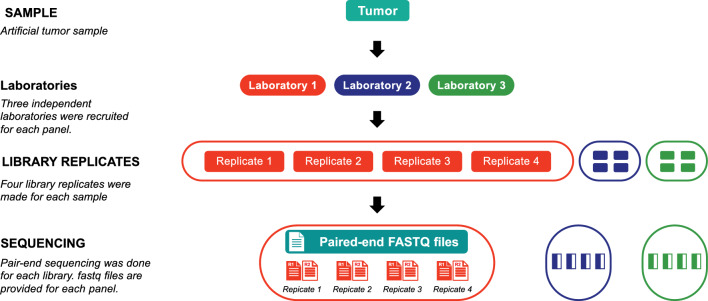
Illustration for dataset. Instead of 4 library replicates per laboratory, there were only three replicates per laboratory for Oncopanel X.

The challenge oncopanel data was created from an artificial tumor sample from the equal mass mixture of genomic DNA from ten Universal Human Reference RNAs (UHRR) cancer cell-lines by Agilent Technologies. The dataset for the challenge was a subset of the oncopanel sequencing dataset^21^ generated in the SEQC2 Oncopanel Sequencing Study. Data from three oncopanels were selected, including AGL (Agilent ClearSeq Comprehensive Cancer Panel v2, Oncopanel A), BRP (Burning Rock DX OncoScreen Plus, Oncopanel B), and ILM (Illumina TruSight Tumor 170, Oncopanel X). The targeted size of AGL, BRP and ILM was 7625, 1631, and 527 kbps, respectively. Three independent laboratories were recruited for each panel. The artificial tumor sample was sent to all laboratories and four library replicates were made at each laboratory. In total, 12 libraries (3 laboratories × 4 library replicates) were created for Oncopanels A and B, 9 libraries (3 laboratories × 3 library replicates) were created for Oncopanel X, and then paired-end sequenced on an Illumina sequencing platform (Fig. 7). The average read counts for Oncopanel A, B, and X were 134, 45, and 85 million read-pairs (details for each library replicate can be found in Supplementary Table 11–13). More information about the oncopanels and the experiments can be found in the related data descriptor^21^.

Metadata for Oncopanels A, B, and X is shown in Supplementary Table 11–13, which contains information about laboratories, library replicates, and filenames. Please note that the UMI barcode for the LAB2 data of Oncopanel A is BLINDED intending to obtain results for NOT using UMI. For the LAB1 and LAB3 data of Oncopanel A, participants are free to choose whether or not to use the UMI information in the pipeline development. All data is available for download from the precisionFDA challenge website (https://precision.fda.gov/challenges/21 and https://precision.fda.gov/challenges/22). Please note that this folder also includes the associated BED files for each dataset. For participants to verify that files have been downloaded correctly, the md5 checksum files were also provided.

### Submission collection and removal of identification information

Each team (or individual) was allowed to submit up to 3 pipeline entries (i.e., set of results). Variant calling should be specified to be in hg19 coordinates. Data processing and pipeline summary was submitted on a voluntary basis. All the submissions were processed by the precisionFDA to mask the identification information by excluding team names and any information that may expose the identity to the evaluation team at NCTR. The identity was only released to NCTR after the completion of performance evaluation and identification of top performers.

**Figure 8:**
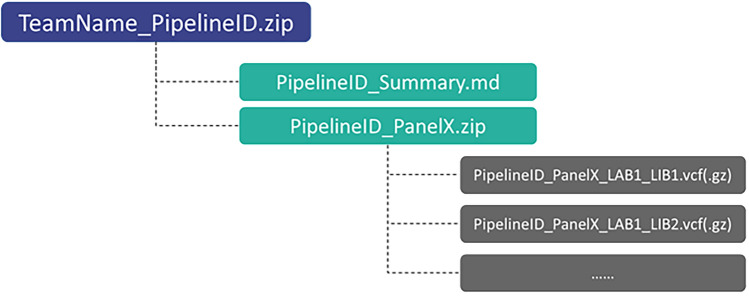
Illustration for organization of submission files.

In each submission (submitted as a zip file, file structure is shown in Fig. 8), variant calling results in VCF format version 4.2 are expected. Submission from two teams, who submitted in the wrong format were excluded from the evaluation.

### Use a known set of indels to assess the performance of indel calling

A known set of indels was used to assess the performance of the submissions. The known set contains two parts, one part is from the KnownPositives set published in our previous study^16^. The other part is from our recent study^22^ to extend the known indel set to the COSMIC gene regions. We created BED files for each oncopanel to contain the regions where the known indel set was studied and the oncopanel covers. There were 267 known indels falling in the targeted regions of the three oncopanels, and more specifically, 262 in Oncopanel A, 72 in Oncopanel B, and 30 in Oncopanel X. Among the 267 known indels, 92 were insertions and 175 were deletions. The length of insertions ranged from 1 to 15 bases, with the majority (90%) being 5 bases or less. The length of deletions ranged from 1 to 36, with the majority (87%) being 5 bases or less. The VAF of the indels ranged from 0.5 to 100%, with the majority (82%) having a VAF below 20%. Details can be found in Supplementary Table 14.

**Figure 9:**
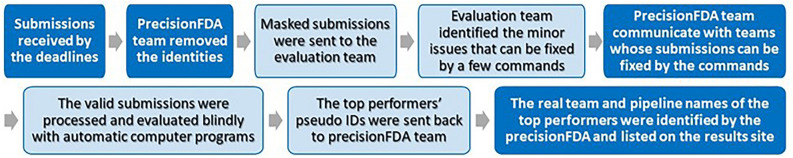
The workflow of performance evaluation.

Figure 9 shows the workflow of evaluation. Submissions received by the deadlines were eligible for evaluation. The precisionFDA team removed the identities with mask-codes before the submissions were sent to the NCTR evaluation team. The evaluations team identified the formatting and other issues that can be fixed by simple commands or widely used tools and sent feedback to the precisionFDA team. The precisionFDA team then contacted the participants for the consents or solutions for correcting their results, and then sent back to the evaluation team. The evaluation team was kept identity-blinded during the whole process until the final ranking results were published.

All valid submissions were format-corrected and evaluated by Linux shell scripts (GNU bash, version 4.2.46) developed by the evaluation team. To better compare the indels from the submissions against our benchmarking indel set, RTG vcfeval tool^19^ (version 3.12.1) was used to determine the true positive indels if they match with any indel in the benchmark indel set. The evaluation was done within each Oncopanel’s evaluation regions. As designed, two libraries, one from Panel A and the other from Panel X, which have relatively low sequencing depth, were excluded from the evaluation, but were used for our own interest. The performance scores were calculated and summarized by Python (version 3.6.15, packaged by conda-forge) and R (version 4.1.3) scripts developed by the evaluation team. Three measures (i.e., recall, precision, and F1 -score) were used for scoring the submissions. The mean over the replicates of each panel, excluding the low-sequencing-depth libraries, was taken for each measure to represent the performance of each submission. Pseudo codes for the evaluation can be found in the Supplemental materials.

$$recall= \frac{number \; of \; true \; positives \; reported \; by \; the \; pipeline}{number \; of \; benchmarking \; indels \; within \; pane{l}{\prime}s \; evaluation \; regions}$$

$$precision= \frac{number \; of \; true \; positives \; reported \; by \; the \; pipeline}{number \; of \; calls \; in \; a \; submission \; within \; the \; pane{l}{\prime}s \; evaluation \; region}$$

$${F}_{1}=2\times \frac{precision \times recall}{ precision+ recall}$$

### Statistical model for performance evaluation

To test whether certain pipelines (listed as “Pipeline2” in the Supplementary Tables 1–9) exhibit similar performance to the top performer (shown as “Pipeline1”) for each oncopanel, we employed a statistical model to assess whether two pipelines are significantly different. This analysis was repeated for each performance metric—recall, precision and F1 score. There were three laboratories and four libraries withing each laboratory, resulting in 12 (3 laboratories × 4 library replicates) combinations for Oncopanel A and B, or 9 (3 laboratories × 3 library replicates) combinations for Oncopanel X. There were also three metrics (Recall, Precision, or F1-score) adopted to assess the performance of each pipeline. Fitting a model to describe the data for comparing the performance metrics among the pipelines, we let y_ijk_ denote the response variable as one of the performance metrics from a pipeline i that was run in a laboratory j using a library k of jth laboratory. The equation of the model is:

$$y_{ijk} = \mu + \tau_{i} + a_{j} + b_{k(j)} + e_{ijk} ,$$

where $\mu + \tau_{i}$ is a fixed parameter representing the mean of the ith pipeline, *i* = 1,…,38. *a*_*j*_ is the effect of jth randomly selected laboratory, *j* = 1, …, 3. *b*_*k*(*j*)_ is the effect of the kth randomly selected library from the jth laboratory, *k* = 1, …, 4. *e*_*ijk*_ is the random error associated with the model.

These are mixed models containing both fixed effects and random effects. The models were implemented in SAS 9.4 to calculate the estimates of the least-square means for the comparisons. Given the large number of comparisons, the p-values computed from the model resulting from the comparisons were adjusted using the Bonferroni multiple comparison procedure to control the overall type 1 error rate for a significance level of 0.05. To assess the variability in the performance metric, we also calculated the CV as the percentage of the standard deviation of the mean for each pipeline.

### Annotation the indels using SnpEff

The indels from both the known indel set and the submitted results were annotated using SnpEff^23^ (version 5.0e) with HGVS annotation (http://www.hgvs.org), where “HGVS.c” and ”HGVS.p” from the annotation results by SnpEff were used to determine whether an indel could be annotated.

### Pipeline summary provided by some participants

Three top performers and two other participants in the best performing cluster kindly shared the summary information of their pipelines (see Supplementary Information).

### Supplementary Information

Supplementary Information 1.

Supplementary Information 2.

## Data Availability

All the data used in this challenge is hosted on the precisionFDA website. The data is open to the public upon registration on the precisionFDA website. Detailed information can be found on the webpages of the challenge (https://precision.fda.gov/challenges/21 and https://precision.fda.gov/challenges/22).
