# Supplementary material for: Towards accurate indel calling for oncopanel sequencing through an international pipeline competition at precisionFDA
Source: Sci Rep. 2024 Apr 8;14:8165. doi: 10.1038/s41598-024-58573-y (PMC11001604; doi:10.1038/s41598-024-58573-y)
Supplement: Supplementary file 1 — Supplementary Information 1. [file 41598_2024_58573_MOESM1_ESM.docx]

**SUPPLEMANTAL MATERIALS**

**Towards accurate indel calling for oncopanel sequencing through an international pipeline competition at precisionFDA**

Binsheng Gong^1^, Samir Lababidi^2^, Rebecca Kusko^3^, Khaled Bouri^4^, Sarah Prezek^5^, Vishal Thovarai^5^, Anish Prasanna^5^, Ezekiel J. Maier^5^, Mahdi Golkaram^6^, Xingqiang Sun^7^, Konstantinos Kyriakidis^8^, João Paulo Kitajima^9^, Sayed Mohammad Ebrahim Sahraeian^10^, Yunfei Guo^10^, Elaine Johanson^2^, Wendell Jones^11^, Weida Tong^1^, Joshua Xu^1*^

**Supplemental Tables**

**Supplemental Table1 (Panel A/Recall): Top pipelines in MEAN descending order value**

| **Pipeline1** | **Pipeline2** | **MEAN Pipeline2** | **%CV Pipeline2** | **Nominal P- Value*** | **Adjusted P-Value*** |
| --- | --- | --- | --- | --- | --- |
| 9FOTT_PH431  Mean=0.9027  %CV=3.48 | 10BL6_1CUWG | 0.89663 | 5.905 | 0.7244 | 1.000 |
|  | CB800_A3SSK | 0.87023 | 2.618 | 0.0589 | 1.000 |
|  | XEMVH_N9YIH | 0.86609 | 5.216 | 0.0333 | 1.000 |
|  | 9FOTT_F1JE0 | 0.84765 | 7.903 | 0.0014 | 0.9970 |
|  | CX4OQ_A0SUG | 0.84288 | 2.971 | 0.0005 | 0.3745 |
|  | HKK8M_06A6Q | 0.83365 | 6.072 | < .0001 | 0.0468 |

* P-Value is for the difference in means between the pipelines from the mixed-effect model.

Adjusted P-Value is based on the Bonferroni multiple adjustment procedure.

Note: The pipeline with highest mean value was shown as “Pipeline1”, and other pipelines were listed in the Column “Pipeline2”, in the descending order of their mean performance value. We only listed the insignificantly different pipelines (i.e., adjusted p-value ≥ 0.05) and the next pipeline that became significantly different (i.e., adjusted p-value < 0.05) in comparison with the top performer. This implied that all the other pipelines not listed were significant different than the top performer “Pipeline1” by the chosen performance metric. This note explains the results in Supplemental Table 1-9.

**Supplemental Table2 (Panel A/Precision): Top pipelines in MEAN descending order value**

| **Pipeline1** | **Pipeline2** | **MEAN Pipeline2** | **%CV Pipeline2** | **Nominal P- Value*** | **Adjusted P-Value*** |
| --- | --- | --- | --- | --- | --- |
| CX4OQ_10897  Mean=0.8926  %CV=5.2882 | XF9RY_4TAIK | 0.88355 | 3.2647 | 0.6261 | 1.000 |
|  | MUN3D_N0YLH | 0.87351 | 2.2605 | 0.2883 | 1.000 |
|  | TDV43_MGFRE | 0.84669 | 2.0148 | 0.0096 | 1.000 |
|  | TDV43_T31KI | 0.84399 | 1.9959 | 0.0061 | 1.000 |
|  | F1VU9_0LSMH | 0.81482 | 10.3482 | < .0001 | 0.0086 |

* P-Value is for the difference in means between the pipelines from the mixed-effect model.

Adjusted P-Value is based on the Bonferroni multiple adjustment procedure.

**Supplemental Table3 (Panel A/F1-Score): Top pipelines in MEAN descending order value**

| **Pipeline1** | **Pipeline2** | **MEAN Pipeline2** | **%CV Pipeline2** | **Nominal P- Value*** | **Adjusted P-Value*** |
| --- | --- | --- | --- | --- | --- |
| XEMVH_N9YIH  Mean=0.81689  %CV=2.4066  ` | TDV43_T31KI | 0.77201 | 3.8843 | 0.0018 | 1.000 |
|  | 9FOTT_F1JE0 | 0.76993 | 3.8530 | 0.0011 | 0.7762 |
|  | 79KK7_UE73P | 0.76954 | 3.0982 | 0.0010 | 0.7052 |
|  | 79KK7_PSUDJ | 0.76899 | 3.3793 | 0.0009 | 0.6164 |
|  | XEMVH_FT0I6 | 0.76892 | 2.3209 | 0.0009 | 0.6063 |
|  | TDV43_MGFRE | 0.76793 | 5.9036 | 0.0007 | 0.4738 |
|  | 79KK7_GDGS2 | 0.76210 | 3.7093 | 0.0001 | 0.1025 |
|  | R6CG9_3G1YB | 0.75199 | 9.1949 | <.0001 | 0.0052 |

* P-Value is for the difference in means between the pipelines from the mixed-effect model.

Adjusted P-Value is based on the Bonferroni multiple adjustment procedure.

**Supplemental Table4 (Panel B/Recall): Top pipelines in MEAN descending order value**

| **Pipeline1** | **Pipeline2** | **MEAN Pipeline2** | **%CV Pipeline2** | **Nominal P- Value*** | **Adjusted P-Value*** |
| --- | --- | --- | --- | --- | --- |
| XEMVH_N9YIH  MEAN=0.92708  %CV=0.6776 | 9FOTT_PH431 | 0.90162 | 1.2215 | 0.0015 | 1.000 |
|  | 10BL6_1CUWG | 0.90046 | 2.0624 | 0.0009 | 0.7519 |
|  | CB800_A3SSK | 0.89699 | 1.3941 | 0.0002 | 0.1504 |
|  | AWJ0W_AXN3J | 0.89120 | 2.3806 | <.0001 | 0.0072 |

* P-Value is for the difference in means between the pipelines from the mixed-effect model.

Adjusted P-Value is based on the Bonferroni multiple adjustment procedure.

**Supplemental Table5 (Panel B/Precision): Top pipelines in MEAN descending order value**

| **Pipeline1** | **Pipeline2** | **MEAN Pipeline2** | **%CV Pipeline2** | **Nominal P- Value*** | **Adjusted P-Value*** |
| --- | --- | --- | --- | --- | --- |
| CX4OQ_10897  Mean=0.98958  %CV=3.6464 | XF9RY_4TAIK | 0.90555 | 3.6464 | <.0001 | 0.0002 |

* P-Value is for the difference in means between the pipelines from the mixed-effect model.

Adjusted P-Value is based on the Bonferroni multiple adjustment procedure.

**Supplemental Table6 (Panel B/F1-Score): Top pipelines in MEAN descending order value**

| **Pipeline1** | **Pipeline2** | **MEAN Pipeline2** | **%CV Pipeline2** | **Nominal P- Value*** | **Adjusted P-Value*** |
| --- | --- | --- | --- | --- | --- |
| R6CG9_3G1YB  Mean=0.7876  %CV=1.75048  ` | AWJ0W_F2MJQ | 0.78723 | 2.46887 | 0.9879 | 1.000 |
|  | 79KK7_GDGS2 | 0.78301 | 0.88816 | 0.6182 | 1.000 |
|  | 79KK7_UE73P | 0.77839 | 0.73998 | 0.3041 | 1.000 |
|  | 79KK7_PSUDJ | 0.77710 | 0.77843 | 0.2402 | 1.000 |
|  | TDV43_T31KI | 0.77401 | 2.57842 | 0.1268 | 1.000 |
|  | TDV43_MGFRE | 0.76815 | 2.70218 | 0.0282 | 1.000 |
|  | R6CG9_8PFS8 | 0.75983 | 2.08637 | 0.0017 | 1.000 |
|  | TDV43_H9B56 | 0.74983 | 2.30081 | <.0001 | 0.0172 |

* P-Value is for the difference in means between the pipelines from the mixed-effect model.

Adjusted P-Value is based on the Bonferroni multiple adjustment procedure.

**Supplemental Table7 (Panel X/Recall): Top pipelines in MEAN descending order value**

| **Pipeline1** | **Pipeline2** | **MEAN Pipeline2** | **%CV Pipeline2** | **Nominal P- Value*** | **Adjusted P-Value*** |
| --- | --- | --- | --- | --- | --- |
| 6JDGL_E5CEL  Mean=0.775  %CV=3.0413   \|  \|  \|  \| \| --- \| --- \| --- \| \|  \|  \|  \| | 9FOTT_PH431 | 0.77037 | 29.5409 | 0.2825 | 1.000 |
|  | 9FOTT_F1JE0 | 0.75185 | 31.0433 | 0.4953 | 1.000 |
|  | 10BL6_1CUWG | 0.73704 | 32.4908 | 0.7135 | 1.000 |
|  | CX4OQ_A0SUG | 0.73704 | 30.7940 | 0.7135 | 1.000 |
|  | XEMVH_N9YIH | 0.72963 | 30.8540 | 0.8338 | 1.000 |
|  | 79KK7_GDGS2 | 0.72593 | 32.3698 | 0.8957 | 1.000 |
|  | 6JDGL_T2NDG | 0.70370 | 30.5558 | 0.7329 | 1.000 |
|  | F1VU9_0LSMH | 0.69630 | 28.5678 | 0.6180 | 1.000 |
|  | 79KK7_PSUDJ | 0.69259 | 33.9277 | 0.5637 | 1.000 |
|  | 79KK7_UE73P | 0.69259 | 33.9277 | 0.5637 | 1.000 |
|  | CB800_A3SSK | 0.68889 | 30.3144 | 0.5118 | 1.000 |
|  | TDV43_H9B56 | 0.66667 | 33.8194 | 0.2596 | 1.000 |
|  | TDV43_MGFRE | 0.66667 | 33.8194 | 0.2596 | 1.000 |
|  | TDV43_T31KI | 0.66667 | 33.8194 | 0.2596 | 1.000 |
|  | XEMVH_ZT92R | 0.65926 | 29.9957 | 0.1991 | 1.000 |
|  | XEMVH_FT0I6 | 0.65185 | 45.0980 | 0.1496 | 1.000 |
|  | K55XH_DESRS | 0.64815 | 31.9221 | 0.1287 | 1.000 |
|  | R6CG9_3G1YB | 0.62963 | 32.8965 | 0.0562 | 1.000 |
|  | R6CG9_8PFS8 | 0.62963 | 32.8965 | 0.0562 | 1.000 |
|  | CB800_ULBET | 0.62593 | 30.4504 | 0.0469 | 1.000 |
|  | HKK8M_06A6Q | 0.62222 | 33.7755 | 0.0389 | 1.000 |
|  | HKK8M_1YT7R | 0.62222 | 33.7755 | 0.0389 | 1.000 |
|  | LYZOZ_7L1OK | 0.58889 | 41.2082 | 0.0057 | 1.000 |
|  | HKK8M_RZYJE | 0.57778 | 31.0683 | 0.0028 | 1.000 |
|  | 78O7G_7AXTN | 0.47407 | 19.5039 | <.0001 | <.0001 |

* P-Value is for the difference in means between the pipelines from the mixed-effect model.

Adjusted P-Value is based on the Bonferroni multiple adjustment procedure.

**Supplemental Table8 (Panel X/Precision): Top pipelines in MEAN descending order value**

| **Pipeline1** | **Pipeline2** | **MEAN Pipeline2** | **%CV Pipeline2** | **Nominal P- Value*** | **Adjusted P-Value*** |
| --- | --- | --- | --- | --- | --- |
| XF9RY_4TAIK  Mean=0.7487  %CV=8.7255 | TDV43_MGFRE | 0.74587 | 19.0821 | 0.9288 | 1.000 |
|  | TDV43_T31KI | 0.71742 | 22.6446 | 0.3198 | 1.000 |
|  | MUN3D_N0YLH | 0.71394 | 33.9482 | 0.2689 | 1.000 |
|  | 9FOTT_H8EJ9 | 0.69453 | 16.4649 | 0.0853 | 1.000 |
|  | 79KK7_UE73P | 0.66569 | 18.8565 | 0.0086 | 1.000 |
|  | TDV43_H9B56 | 0.65684 | 24.2135 | 0.0037 | 1.000 |
|  | 79KK7_PSUDJ | 0.62103 | 21.5533 | <.0001 | 0.0385 |

* P-Value is for the difference in means between the pipelines from the mixed-effect model.

Adjusted P-Value is based on the Bonferroni multiple adjustment procedure.

**Supplemental Table9 (Panel X/F1-Score): Top pipelines in MEAN descending order value**

| **Pipeline1** | **Pipeline2** | **MEAN Pipeline2** | **%CV Pipeline2** | **Nominal P- Value*** | **Adjusted P-Value*** |
| --- | --- | --- | --- | --- | --- |
| TDV43_MGFRE  Mean=0.69327  %CV=31.4617  ` | TDV43_T31KI | 0.68302 | 31.6938 | 0.7653 | 1.000 |
|  | 79KK7_UE73P | 0.66796 | 31.2999 | 0.4613 | 1.000 |
|  | TDV43_H9B56 | 0.65480 | 31.6900 | 0.2631 | 1.000 |
|  | 79KK7_PSUDJ | 0.64601 | 31.3933 | 0.1694 | 1.000 |
|  | 79KK7_GDGS2 | 0.63448 | 29.1748 | 0.0877 | 1.000 |
|  | R6CG9_3G1YB | 0.61290 | 31.9853 | 0.0198 | 1.000 |
|  | R6CG9_8PFS8 | 0.59083 | 32.9084 | 0.0031 | 1.000 |
|  | LYZOZ_7L1OK | 0.55230 | 34.7447 | <.0001 | 0.0328 |

* P-Value is for the difference in means between the pipelines from the mixed-effect model.

Adjusted P-Value is based on the Bonferroni multiple adjustment procedure.

**Supplemental Table 10: The mean and CV values of precision, recall, and F1-score of the pipelines**

| **Oncopanel A** | | | | | | |
| --- | --- | --- | --- | --- | --- | --- |
|  | **Precision** | | **Recall** | | **F1-score** | |
| ***TEAM_PIPELINE*** | ***MEAN*** | ***CV*** | ***MEAN*** | ***CV*** | ***MEAN*** | ***CV*** |
| **10BL6_1CUWG** | 55.41% | 6.86% | **89.66%** | 5.91% | 68.26% | 1.99% |
| 4KJIN_BV60L | 68.64% | 11.68% | 14.47% | 3.46% | 23.84% | 3.52% |
| 4KJIN_J05DX | 71.30% | 9.07% | 13.42% | 2.04% | 22.57% | 3.09% |
| 4KJIN_ZW8IZ | 65.14% | 9.42% | 14.98% | 1.92% | 24.33% | 3.33% |
| 6JDGL_E5CEL | 63.04% | 3.88% | 67.27% | 10.10% | 64.82% | 5.21% |
| 6JDGL_T2NDG | 65.01% | 3.82% | 61.20% | 10.08% | 62.85% | 6.18% |
| 78O7G_7AXTN | 50.91% | 9.55% | 64.41% | 1.82% | 56.78% | 6.43% |
| 78O7G_TOC8H | 62.80% | 6.25% | 56.30% | 2.98% | 59.34% | 4.47% |
| 79KK7_GDGS2 | 70.45% | 2.84% | 83.02% | 4.79% | 76.21% | 3.71% |
| 79KK7_PSUDJ | 72.38% | 2.10% | 82.06% | 4.90% | 76.90% | 3.38% |
| 79KK7_UE73P | 73.26% | 1.63% | 81.33% | 6.85% | 76.95% | 3.10% |
| 9FOTT_F1JE0 | 70.78% | 1.71% | 84.76% | 7.90% | 76.99% | 3.85% |
| 9FOTT_H8EJ9 | 71.30% | 3.58% | 25.13% | 3.23% | 37.15% | 2.87% |
| 9FOTT_PH431 | 44.06% | 10.15% | 90.27% | 3.48% | 59.13% | 7.78% |
| CB800_A3SSK | 44.00% | 8.95% | 87.02% | 2.62% | 58.39% | 6.88% |
| CB800_ULBET | 49.52% | 5.12% | 83.11% | 3.54% | 62.05% | 4.34% |
| CX4OQ_A0SUG | 64.65% | 16.56% | 84.29% | 2.97% | 72.79% | 10.82% |
| CX4OQ_I0897 | 89.21% | 5.29% | 10.69% | 18.34% | 18.99% | 14.96% |
| CX4OQ_UQ9YO | 61.80% | 3.34% | 24.36% | 5.12% | 34.93% | 4.17% |
| F1VU9_0LSMH | 81.48% | 10.35% | 12.88% | 53.71% | 21.41% | 45.77% |
| F1VU9_2PJYP | 77.83% | 8.58% | 23.79% | 17.86% | 36.08% | 11.58% |
| F1VU9_Q18GL | 77.64% | 8.81% | 24.05% | 19.20% | 36.31% | 12.32% |
| HKK8M_06A6Q | 62.27% | 3.19% | 83.37% | 6.07% | 71.23% | 3.83% |
| HKK8M_1YT7R | 66.48% | 4.01% | 79.74% | 9.92% | 72.34% | 6.24% |
| HKK8M_RZYJE | 44.85% | 12.76% | 71.37% | 10.73% | 54.87% | 10.85% |
| K55XH_DESRS | 75.45% | 5.73% | 73.66% | 7.62% | 74.30% | 4.12% |
| LYZOZ_7L1OK | 5.78% | 93.97% | 3.47% | 107.40% | 4.32% | 102.33% |
| MUN3D_N0YLH | 87.35% | 2.26% | 33.97% | 6.76% | 48.87% | 5.14% |
| R6CG9_3G1YB | 80.02% | 1.90% | 71.79% | 15.29% | 75.20% | 9.19% |
| R6CG9_7UK7Q | 70.79% | 2.66% | 76.30% | 16.39% | 72.96% | 9.60% |
| R6CG9_8PFS8 | 75.88% | 8.30% | 72.71% | 13.06% | 74.08% | 10.27% |
| TDV43_H9B56 | 76.22% | 2.45% | 72.14% | 6.84% | 74.00% | 3.51% |
| TDV43_MGFRE | 84.67% | 2.01% | 70.64% | 9.93% | 76.79% | 5.90% |
| TDV43_T31KI | 84.40% | 2.00% | 71.34% | 7.05% | 77.20% | 3.88% |
| XEMVH_FT0I6 | 73.85% | 0.52% | 80.31% | 4.87% | 76.89% | 2.32% |
| **XEMVH_N9YIH** | 77.42% | 1.05% | 86.61% | 5.22% | **81.69%** | 2.41% |
| XEMVH_ZT92R | 60.64% | 9.57% | 64.79% | 13.80% | 61.83% | 6.18% |
| **XF9RY_4TAIK** | **88.35%** | 3.26% | 51.75% | 11.24% | 65.16% | 8.66% |

*(continue)*

| **Oncopanel B** | | | | | | |
| --- | --- | --- | --- | --- | --- | --- |
|  | **Precision** | | **Recall** | | **F1-score** | |
| ***TEAM_PIPELINE*** | ***MEAN*** | ***CV*** | ***MEAN*** | ***CV*** | ***MEAN*** | ***CV*** |
| 10BL6_1CUWG | 32.28% | 5.20% | 90.05% | 2.06% | 47.50% | 3.92% |
| 4KJIN_BV60L | 63.45% | 5.01% | 22.34% | 1.79% | 33.02% | 1.27% |
| 4KJIN_J05DX | 73.09% | 5.74% | 20.83% | 0.00% | 32.41% | 1.26% |
| 4KJIN_ZW8IZ | 63.45% | 5.01% | 22.34% | 1.79% | 33.02% | 1.27% |
| 6JDGL_E5CEL | 30.42% | 6.47% | 78.70% | 4.21% | 43.84% | 5.00% |
| 6JDGL_T2NDG | 31.68% | 8.31% | 69.44% | 4.26% | 43.45% | 6.17% |
| 78O7G_7AXTN | 35.06% | 2.23% | 59.84% | 1.55% | 44.21% | 1.73% |
| 78O7G_TOC8H | 39.55% | 3.76% | 59.49% | 2.19% | 47.50% | 2.65% |
| 79KK7_GDGS2 | 74.23% | 2.02% | 82.87% | 0.83% | 78.30% | 0.89% |
| 79KK7_PSUDJ | 74.29% | 1.89% | 81.48% | 0.84% | 77.71% | 0.78% |
| 79KK7_UE73P | 74.53% | 1.85% | 81.48% | 0.84% | 77.84% | 0.74% |
| 9FOTT_F1JE0 | 43.90% | 4.29% | 86.69% | 1.87% | 58.26% | 2.89% |
| 9FOTT_H8EJ9 | 68.26% | 4.31% | 23.38% | 6.12% | 34.82% | 5.30% |
| 9FOTT_PH431 | 27.86% | 3.79% | 90.16% | 1.22% | 42.55% | 2.82% |
| AWJ0W_AXN3J | 28.71% | 6.32% | 89.12% | 2.38% | 43.38% | 4.54% |
| AWJ0W_F2MJQ | 89.25% | 3.21% | 70.49% | 3.67% | 78.72% | 2.47% |
| AWJ0W_QN0E8 | 84.39% | 7.91% | 34.61% | 10.33% | 49.06% | 9.39% |
| CB800_A3SSK | 31.33% | 5.97% | 89.70% | 1.39% | 46.40% | 4.20% |
| CB800_ULBET | 39.53% | 5.34% | 88.54% | 1.79% | 54.61% | 3.53% |
| CX4OQ_A0SUG | 36.40% | 4.93% | 82.87% | 1.09% | 50.56% | 3.59% |
| CX4OQ_I0897 | 98.96% | 3.65% | 7.87% | 15.66% | 14.55% | 14.39% |
| CX4OQ_UQ9YO | 63.13% | 5.70% | 23.26% | 5.17% | 33.98% | 4.63% |
| F1VU9_0LSMH | 78.29% | 19.22% | 5.32% | 29.08% | 9.90% | 26.78% |
| F1VU9_2PJYP | 71.57% | 13.44% | 20.14% | 24.52% | 31.10% | 19.30% |
| F1VU9_Q18GL | 70.03% | 13.00% | 21.18% | 25.95% | 32.10% | 19.33% |
| HKK8M_06A6Q | 31.72% | 5.65% | 82.87% | 2.18% | 45.86% | 4.40% |
| HKK8M_1YT7R | 44.90% | 4.13% | 81.94% | 2.50% | 57.99% | 3.06% |
| HKK8M_RZYJE | 13.76% | 10.96% | 75.00% | 1.58% | 23.23% | 9.38% |
| K55XH_DESRS | 36.07% | 4.95% | 78.01% | 1.83% | 49.29% | 3.07% |
| LYZOZ_7L1OK | 11.27% | 9.15% | 12.96% | 6.98% | 12.01% | 4.83% |
| MUN3D_N0YLH | 78.36% | 8.42% | 39.58% | 4.10% | 52.52% | 4.44% |
| **R6CG9_3G1YB** | 73.47% | 3.99% | 84.95% | 2.40% | **78.74%** | 1.75% |
| R6CG9_7UK7Q | 60.46% | 4.17% | 87.96% | 1.55% | 71.63% | 2.54% |
| R6CG9_8PFS8 | 68.85% | 4.79% | 84.95% | 2.40% | 75.98% | 2.09% |
| TDV43_H9B56 | 72.13% | 3.97% | 78.13% | 1.54% | 74.98% | 2.30% |
| TDV43_MGFRE | 77.09% | 4.22% | 76.62% | 2.77% | 76.82% | 2.70% |
| TDV43_T31KI | 77.10% | 4.36% | 77.78% | 2.01% | 77.40% | 2.58% |
| XEMVH_FT0I6 | 44.84% | 1.18% | 87.50% | 0.00% | 59.29% | 0.78% |
| **XEMVH_N9YIH** | 38.01% | 2.30% | **92.71%** | 0.68% | 53.91% | 1.74% |
| XEMVH_ZT92R | 42.03% | 7.98% | 69.33% | 3.47% | 52.26% | 5.30% |
| **XF9RY_4TAIK** | **90.56%** | 1.42% | 59.84% | 2.88% | 72.04% | 1.91% |

*(continue)*

| **Oncopanel X** | | | | | | |
| --- | --- | --- | --- | --- | --- | --- |
|  | **Precision** | | **Recall** | | **F1-score** | |
| ***TEAM_PIPELINE*** | ***MEAN*** | ***CV*** | ***MEAN*** | ***CV*** | ***MEAN*** | ***CV*** |
| 10BL6_1CUWG | 37.32% | 25.88% | 73.70% | 32.49% | 49.21% | 29.56% |
| 4KJIN_J05DX | 51.39% | 26.11% | 27.78% | 24.00% | 35.73% | 23.29% |
| 4KJIN_ZW8IZ | 46.59% | 21.09% | 31.11% | 21.43% | 37.10% | 20.10% |
| 6JDGL_E5CEL | 21.04% | 15.83% | 77.50% | 3.04% | 33.00% | 12.92% |
| 6JDGL_T2NDG | 17.70% | 30.70% | 70.37% | 30.56% | 28.20% | 30.46% |
| 78O7G_7AXTN | 27.63% | 15.18% | 47.41% | 19.50% | 34.87% | 16.85% |
| 78O7G_TOC8H | 30.40% | 25.32% | 41.48% | 30.63% | 34.99% | 27.86% |
| 79KK7_GDGS2 | 57.35% | 21.64% | 72.59% | 32.37% | 63.45% | 29.17% |
| 79KK7_PSUDJ | 62.10% | 21.55% | 69.26% | 33.93% | 64.60% | 31.39% |
| 79KK7_UE73P | 66.57% | 18.86% | 69.26% | 33.93% | 66.80% | 31.30% |
| **9FOTT_F1JE0** | 36.76% | 29.55% | **75.19%** | 31.04% | 49.22% | 29.96% |
| 9FOTT_H8EJ9 | 69.45% | 16.46% | 17.04% | 18.16% | 27.20% | 16.72% |
| 9FOTT_PH431 | 22.37% | 27.20% | 77.04% | 29.54% | 34.51% | 27.71% |
| CB800_A3SSK | 30.75% | 27.16% | 68.89% | 30.31% | 42.37% | 28.37% |
| CB800_ULBET | 38.17% | 22.58% | 62.59% | 30.45% | 47.19% | 26.64% |
| CX4OQ_A0SUG | 28.17% | 34.73% | 73.70% | 30.79% | 40.56% | 32.86% |
| CX4OQ_UQ9YO | 59.30% | 12.42% | 20.00% | 22.05% | 29.76% | 19.08% |
| F1VU9_0LSMH | 22.89% | 27.71% | 69.63% | 28.57% | 34.28% | 27.57% |
| F1VU9_2PJYP | 51.32% | 24.54% | 23.33% | 30.30% | 31.66% | 27.57% |
| F1VU9_Q18GL | 51.96% | 23.33% | 24.07% | 31.56% | 32.40% | 27.61% |
| HKK8M_06A6Q | 38.26% | 31.59% | 62.22% | 33.78% | 46.78% | 32.79% |
| HKK8M_1YT7R | 38.46% | 31.33% | 62.22% | 33.78% | 46.95% | 32.69% |
| HKK8M_RZYJE | 13.88% | 28.01% | 57.78% | 31.07% | 22.34% | 28.77% |
| K55XH_DESRS | 33.29% | 28.59% | 64.81% | 31.92% | 43.80% | 29.93% |
| LYZOZ_7L1OK | 53.38% | 29.96% | 58.89% | 41.21% | 55.23% | 34.74% |
| MUN3D_N0YLH | 71.39% | 33.95% | 32.22% | 34.70% | 44.15% | 34.42% |
| R6CG9_3G1YB | 60.40% | 31.37% | 62.96% | 32.90% | 61.29% | 31.99% |
| R6CG9_7UK7Q | 52.51% | 25.92% | 30.00% | 88.19% | 34.75% | 66.35% |
| R6CG9_8PFS8 | 56.21% | 33.85% | 62.96% | 32.90% | 59.08% | 32.91% |
| TDV43_H9B56 | 65.68% | 24.21% | 66.67% | 33.82% | 65.48% | 31.69% |
| **TDV43_MGFRE** | **74.59%** | 19.08% | 66.67% | 33.82% | **69.33%** | 31.46% |
| TDV43_T31KI | 71.74% | 22.64% | 66.67% | 33.82% | 68.30% | 31.69% |
| XEMVH_FT0I6 | 27.45% | 37.91% | 65.19% | 45.10% | 38.46% | 40.74% |
| XEMVH_N9YIH | 31.28% | 29.05% | 72.96% | 30.85% | 43.73% | 29.60% |
| XEMVH_ZT92R | 37.56% | 23.85% | 65.93% | 30.00% | 47.59% | 26.87% |
| XF9RY_4TAIK | 74.87% | 8.73% | 15.56% | 21.43% | 25.62% | 19.81% |

Note: The bolded pipelines are the top performers in the bolded category recognized in this precisionFDA challenge.

**Supplemental Table 11**: Oncopanel A metadata

| Laboratory | Library Replicate | Total Read-pairs (million) | High Quality Read (%, Phred>=30) | Left Read | UMI Barcode | Right Read |
| --- | --- | --- | --- | --- | --- | --- |
| Lab 1 | Lib 1 | 171.4 | 95 | PanelA_LAB1_LIB1_R1.fastq.gz | PanelA_LAB1_LIB1_R2.fastq.gz | PanelA_LAB1_LIB1_R3.fastq.gz |
| Lab 1 | Lib 2 | 179.2 | 94 | PanelA_LAB1_LIB2_R1.fastq.gz | PanelA_LAB1_LIB2_R2.fastq.gz | PanelA_LAB1_LIB2_R3.fastq.gz |
| Lab 1 | Lib 3 | 151.7 | 95 | PanelA_LAB1_LIB3_R1.fastq.gz | PanelA_LAB1_LIB3_R2.fastq.gz | PanelA_LAB1_LIB3_R3.fastq.gz |
| Lab 1 | Lib 4 | 169.4 | 95 | PanelA_LAB1_LIB4_R1.fastq.gz | PanelA_LAB1_LIB4_R2.fastq.gz | PanelA_LAB1_LIB4_R3.fastq.gz |
| Lab 2 | Lib 1 | 94.6 | 97 | PanelA_LAB2_LIB1_R1.fastq.gz | PanelA_LAB2_LIB1_R2.fastq.gz | PanelA_LAB2_LIB1_R3.fastq.gz |
| Lab 2 | Lib 2 | 104.5 | 96 | PanelA_LAB2_LIB2_R1.fastq.gz | PanelA_LAB2_LIB2_R2.fastq.gz | PanelA_LAB2_LIB2_R3.fastq.gz |
| Lab 2 | Lib 3 | 27.6 | 97 | PanelA_LAB2_LIB3_R1.fastq.gz | PanelA_LAB2_LIB3_R2.fastq.gz | PanelA_LAB2_LIB3_R3.fastq.gz |
| Lab 2 | Lib 4 | 116.4 | 97 | PanelA_LAB2_LIB4_R1.fastq.gz | PanelA_LAB2_LIB4_R2.fastq.gz | PanelA_LAB2_LIB4_R3.fastq.gz |
| Lab 3 | Lib 1 | 134.5 | 95 | PanelA_LAB3_LIB1_R1.fastq.gz | PanelA_LAB3_LIB1_R2.fastq.gz | PanelA_LAB3_LIB1_R3.fastq.gz |
| Lab 3 | Lib 2 | 177.1 | 94 | PanelA_LAB3_LIB2_R1.fastq.gz | PanelA_LAB3_LIB2_R2.fastq.gz | PanelA_LAB3_LIB2_R3.fastq.gz |
| Lab 3 | Lib 3 | 149.1 | 95 | PanelA_LAB3_LIB3_R1.fastq.gz | PanelA_LAB3_LIB3_R2.fastq.gz | PanelA_LAB3_LIB3_R3.fastq.gz |
| Lab 3 | Lib 4 | 134.4 | 96 | PanelA_LAB3_LIB4_R1.fastq.gz | PanelA_LAB3_LIB4_R2.fastq.gz | PanelA_LAB3_LIB4_R3.fastq.gz |

**Supplemental Table 12**: Oncopanel B metadata

| Laboratory | Library Replicate | Total Read-pairs (million) | High Quality Read (%, Phred>=30) | Left Read | Right Read |
| --- | --- | --- | --- | --- | --- |
| Lab 1 | Lib 1 | 40.3 | 97 | PanelB_LAB1_LIB1_R1.fastq.gz | PanelB_LAB1_LIB1_R2.fastq.gz |
| Lab 1 | Lib 2 | 49.4 | 97 | PanelB_LAB1_LIB2_R1.fastq.gz | PanelB_LAB1_LIB2_R2.fastq.gz |
| Lab 1 | Lib 3 | 37.3 | 97 | PanelB_LAB1_LIB3_R1.fastq.gz | PanelB_LAB1_LIB3_R2.fastq.gz |
| Lab 1 | Lib 4 | 53.5 | 97 | PanelB_LAB1_LIB4_R1.fastq.gz | PanelB_LAB1_LIB4_R2.fastq.gz |
| Lab 2 | Lib 1 | 48.6 | 97 | PanelB_LAB2_LIB1_R1.fastq.gz | PanelB_LAB2_LIB1_R2.fastq.gz |
| Lab 2 | Lib 2 | 48.0 | 97 | PanelB_LAB2_LIB2_R1.fastq.gz | PanelB_LAB2_LIB2_R2.fastq.gz |
| Lab 2 | Lib 3 | 36.5 | 97 | PanelB_LAB2_LIB3_R1.fastq.gz | PanelB_LAB2_LIB3_R2.fastq.gz |
| Lab 2 | Lib 4 | 38.2 | 97 | PanelB_LAB2_LIB4_R1.fastq.gz | PanelB_LAB2_LIB4_R2.fastq.gz |
| Lab 3 | Lib 1 | 52.2 | 95 | PanelB_LAB3_LIB1_R1.fastq.gz | PanelB_LAB3_LIB1_R2.fastq.gz |
| Lab 3 | Lib 2 | 48.4 | 96 | PanelB_LAB3_LIB2_R1.fastq.gz | PanelB_LAB3_LIB2_R2.fastq.gz |
| Lab 3 | Lib 3 | 44.0 | 95 | PanelB_LAB3_LIB3_R1.fastq.gz | PanelB_LAB3_LIB3_R2.fastq.gz |
| Lab 3 | Lib 4 | 39.9 | 96 | PanelB_LAB3_LIB4_R1.fastq.gz | PanelB_LAB3_LIB4_R2.fastq.gz |

**Supplemental Table 13**: Oncopanel X metadata

| Laboratory | Library Replicate | Total Read-pairs (million) | High Quality Read (%, Phred>=30) | Left Read | Right Read |
| --- | --- | --- | --- | --- | --- |
| Lab 1 | Lib 1 | 98.0 | 85 | PanelX_LAB1_LIB1_R1.fastq.gz | PanelX_LAB1_LIB1_R2.fastq.gz |
| Lab 1 | Lib 2 | 95.1 | 88 | PanelX_LAB1_LIB2_R1.fastq.gz | PanelX_LAB1_LIB2_R2.fastq.gz |
| Lab 1 | Lib 3 | 95.3 | 85 | PanelX_LAB1_LIB3_R1.fastq.gz | PanelX_LAB1_LIB3_R2.fastq.gz |
| Lab 2 | Lib 1 | 77.6 | 84 | PanelX_LAB2_LIB1_R1.fastq.gz | PanelX_LAB2_LIB1_R2.fastq.gz |
| Lab 2 | Lib 2 | 69.7 | 90 | PanelX_LAB2_LIB2_R1.fastq.gz | PanelX_LAB2_LIB2_R2.fastq.gz |
| Lab 2 | Lib 3 | 84.0 | 89 | PanelX_LAB2_LIB3_R1.fastq.gz | PanelX_LAB2_LIB3_R2.fastq.gz |
| Lab 3 | Lib 1 | 85.8 | 89 | PanelX_LAB3_LIB1_R1.fastq.gz | PanelX_LAB3_LIB1_R2.fastq.gz |
| Lab 3 | Lib 2 | 84.0 | 87 | PanelX_LAB3_LIB2_R1.fastq.gz | PanelX_LAB3_LIB2_R2.fastq.gz |
| Lab 3 | Lib 3 | 75.9 | 91 | PanelX_LAB3_LIB3_R1.fastq.gz | PanelX_LAB3_LIB3_R2.fastq.gz |

**Supplemental Table 14**: Detailed information of 267 known indels utilized in this precisionFDA challenge

See the Microsoft Excel file Suppl_Table_14.xlsx

**Supplemental Figures**


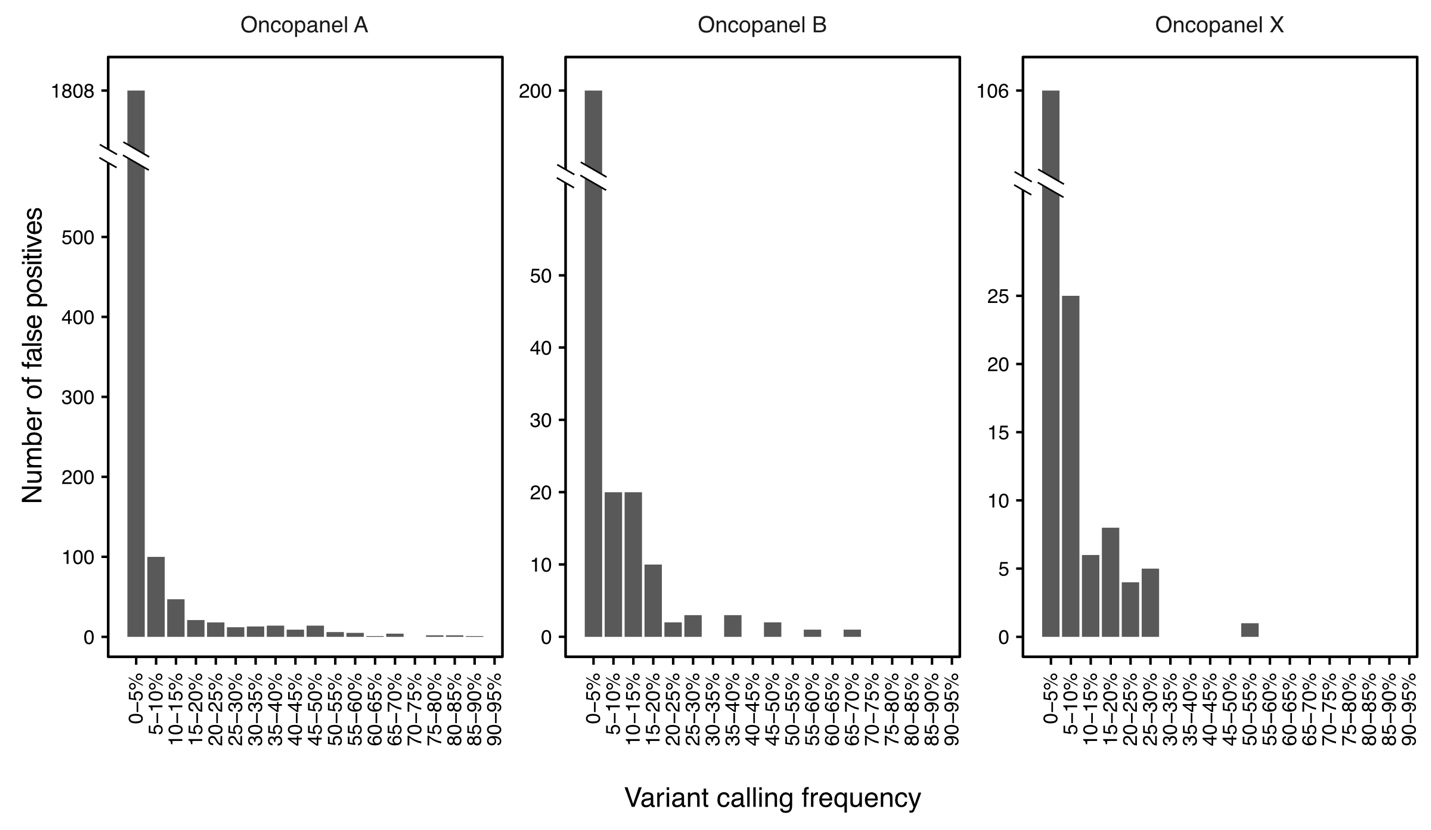


**Supplemental Figure 1**: The number of false positives called at different frequency by the library replicate – pipeline combinations (408 for Oncopanel A, 264 for Oncopanel B, and 153 for Oncopanel X). X-axis shows the frequency buckets, and y-axis is the number of false positives called in each frequency bucket.


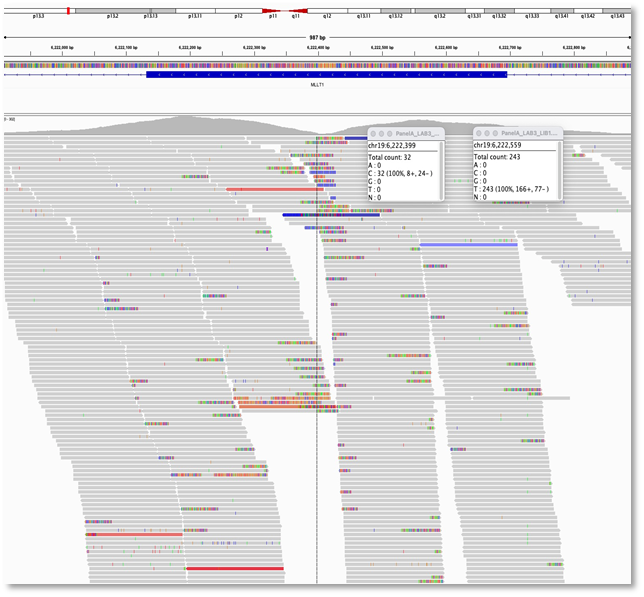


Supplemental Figure 2: Visualization of mapped reads around a “false negative”.


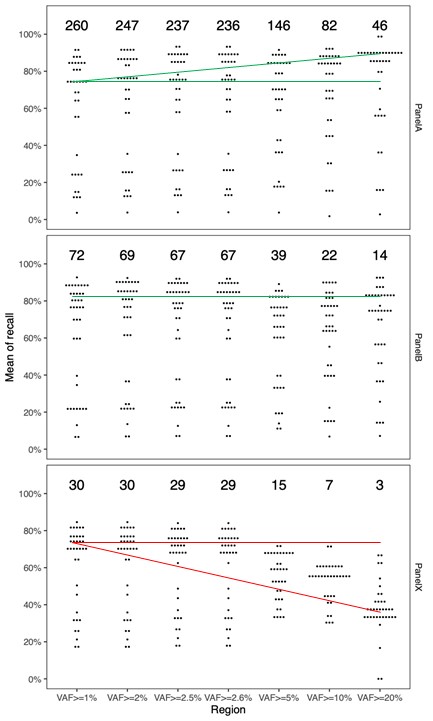


Supplemental Figure 3: The recall was changed after applying different variant allele frequency (VAF) cut-offs.

**Pseudo codes for the evaluation**

**Step 1: Rename the submissions to mask the information about the participants and pipelines**

This step was done manually by the precisionFDA support team

**Step 2: Check and correct the submitted VCF files**

Set submission_folder to “precisionFDA/masked_submission”

Set corrected_folder to “precisionFDA/corrected”

For each VCF_file in submission_folder:

Correct the file names by replacing the Greek alphabet “Χ” with English letter “X”

Exclude submissions based on reference genome release other than hg19

Check and fix VCF header

Check and fix VCF fields

Remove empty lines

Replace continuous multiple spaces into one TAB

Add “chr” to the contig names if contigs do not start with “chr”

Remove record lines if “ALT” is the same as “REF”

Filter variants by type and keep only the indels

Save the checked and corrected VCF file to corrected_folder

**Step 2: Prepare the known indel sets for the three oncopanels**

Set published_known_indels to "KnownPositives.hg19.vcf.gz"

Set extended_known_indels to "Extended_Indels.hg19.vcf.gz"

Combine published_known_indels and extended_known_indels into known_indels

Set regions_for_published_known_indels to "KnownPositives.hg19.bed"

Set regions_for_extended_known_indels to "Extended_indels.regions.hg19.bed"

Combine regions_for_published_known_indels and regions_for_extended_known_indels

into restricted_regions

Set regions_for_OncopanelA to "PanelA.bed"

Set regions_for_OncopanelB to "PanelB.bed"

Set regions_for_OncopanelX to "PanelX.bed"

For each oncopanel in OncopanelA, OncopanelB, and OncopanelX:

Set panel_restricted_region to "$^1^_restricted.bed"

Set known_indels_for_oncopanel to "known_indels_for_$^1^.vcf.gz"

Intersect regions_for_$^1^ with restricted_regions

and save the result to panel_restricted_region

Filter known_indels by panel_restricted_region

and save the result to known_indels_for_oncopanel

**Step 3: Compare the VCF files from the submissions against known indel set for each oncopanel**

Set RefSDF to "RTG/hg19.sdf"

Set evaluation to "precisionFDA_Challenge/evaluation"

Find all ".vcf.gz" files in subdir and iterate over them:

Determine oncopanel, lab, and lib based on the directory and file name

Set rtg_result to "${evaluation}/${oncopanel}/${submission}/${lab}_${lib}"

Run RTG vcfeval comparing the current VCF with TP_vcf, using RefSDF,

and outputting to rtg_result

Copy the current VCF to rtg_result as “all_calls.vcf”

**Step 4: Count the numbers of records from the comparison results from RTG vcfeval and calculate precision, recall, and F1-score**

For each rtg_result in evaluation and its subdirectories:

Determine oncopanel, lab, and lib based on the names of subdirectories

Set num_all_calls to the total number of unique records in “all_calls.vcf”

Set num_all_known_indels_for_$^1^ to the total number of records

in "known_indels_for_$^1^.vcf.gz"

Set num_known_indels_called to the total number of records in “tp_baseline.vcf”

Set num_positive_calls to the total number of unique records in “tp.vcf”

Check the according VCF files manually

if num_positive_calls equals to num_known_indels_called

Set recall to num_known_indels_called / num_all_known_indels_for_$^1^

Set precision to num_positive_calls / num_all_calls

Set F1_score to 2 * precision * recall / (precision + recall)

Save evaluation, oncopanel, lab, lib, and all values calculated above to Excel file

**SAS codes for the statistical model**

********************************************************************************************

Analysis for NCTR/PrecisionFDA Challenge on OncoPanels

******************************************************************************************** ;

LIBNAME in 'DATASET FOLDER'; *** This is where the SAS datasets are;

ODS HTML CLOSE;

ODS LISTING;

OPTIONS PS=35 LS=150;

******************************************************************************************** ;

*******************************************************

Example - PanelA: Recall

Similar codes for other oncopanels and performance metrics

******************************************************* ;

DATA PANELA;SET in.PANELA;TEAM_PIPELINE=TRIM(TEAM)||'_'||PIPELINE;RUN;

DATA PANELA_II;SET PANELA;LAB=SUBSTR(REPLICATE,1,4);LIB=SUBSTR(REPLICATE,6);RUN;

ODS HTML;

PROC MIXED DATA=PANELA_II;

CLASS TEAM_PIPELINE LAB LIB;

MODEL RECALL=TEAM_PIPELINE;

RANDOM LAB LIB(LAB);

LSMEANS TEAM_PIPELINE / PDIFF ADJUST=BON;

RUN;

ODS HTML CLOSE;

********************************************************* MEANS and CVs;

PROC SORT DATA=PANELA OUT=PANELA1;BY TEAM_PIPELINE;RUN;

PROC UNIVARIATE DATA=PANELA1 NOPRINT;BY TEAM_PIPELINE;VAR RECALL;OUTPUT OUT=PANELA_RECALL MEAN=MEAN CV=CV;RUN;

PROC SORT DATA=PANELA_RECALL;BY DESCENDING MEAN;RUN;

ODS HTML;PROC PRINT DATA=PANELA_RECALL;RUN;ODS HTML CLOSE;

********************************************************* Plotting;

DATA PANELA_RECALL_PLOT;SET PANELA_RECALL;SEQ+1;RUN;

PROC CONTENTS DATA=in.PANELA;RUN;

PROC PRINT DATA=PANELA_RECALL_PLOT;RUN;

proc sql;

create table foo as select unique

SEQ as start,

TEAM_PIPELINE as label

from PANELA_RECALL_PLOT;

quit; run;

PROC PRINT DATA=foo;RUN;

data control; set foo;

fmtname = 'barfmt';

type = 'N';

end = START;

run;

proc format lib=work cntlin=control;

run;

*** MEAN;

goptions reset=all border HTEXT=1.5;

axis1 LABEL=(A=90 'Mean Recall');

axis2 label=NONE VALUE=(H=1 F=SWISSB A=55);

PROC GCHART DATA=PANELA_RECALL_PLOT;

FORMAT SEQ barfmt.;

VBAR SEQ / DISCRETE SUMVAR=MEAN OUTSIDE=SUM

RAXIS=axis1 maxis=axis2;

RUN;

quit;

*** CV;

goptions reset=all border HTEXT=1.5;

axis1 LABEL=(A=90 '%CV Recall');

axis2 label=NONE VALUE=(H=1 F=SWISSB A=55);

PROC GCHART DATA=PANELA_RECALL_PLOT;

FORMAT SEQ barfmt.;

VBAR SEQ / DISCRETE SUMVAR=CV

RAXIS=axis1 maxis=axis2 Ref=10;

RUN;

quit;

********************************************************* ;

**Supplemental Information**

**Pipeline summary provided by some top performers**

**Disclaimers**: The content that follows is provided by a select group of top performers from the associated challenge. It is important to note that these perspectives, while insightful and valuable, are their own and do not necessarily represent or reflect the views, opinions, or positions held in this manuscript or by the organizers of the precisionFDA challenge. These contributions should be considered as supplementary, individual perspectives that may enhance the understanding and interpretation of the research, rather than a consensus viewpoint or official stance. Readers are advised to approach these insights with an open and discerning mind, and consider them as part of a broader discussion rather than definitive conclusions.

Illumina DRAGEN pipeline (Participant ID: TDV43)

The DRAGEN pipeline enables end-to-end processing of genomics data^2^ and has previously outperformed other state-of-the-art tools in detecting germline variants in difficult-to-map regions of the genome^3,4^ DRAGEN can achieve a high accuracy by leveraging: 1) improved alignment to a multi-genome graph reference, which is critical in regions of the genome with high homology,^5^ 2) positional clustering with libraries with high UMI hopping rate, 3) a single (highly accurate) somatic variant caller. Relying on a single variant caller (as opposed to ensemble callers) together with FPGA acceleration enables DRAGEN to achieve significantly shorter computing time.

DRAGEN somatic indel caller inherits GATK 4.0^6^ overall architecture while the core Bayesian genotyping model is based on that of Strelka2.^7^ Furthermore, DRAGEN uses a hidden Markov model that performs read likelihood calculation based on nucleotide error bias and sample-specific estimation of indel error rates as well as probability models of common error modes such as strand bias, orientation bias, and mismapping. Finally, when a paired normal is not available, DRAGEN can attenuate sequencing and library preparation artefacts by estimating systematic noise, based on a panel of unpaired normal samples (preferably prepared using the same assay workflow, coverage, sequencer, targeted enrichment probes). ^8^

Currently, DRAGEN somatic indel caller (in contrast with DRAGEN V4.0 germline variant caller) does not leverage machine learning due to lack of accurate, reliable, and realistic somatic variant calling truth data. However, it is expected as more somatic variant calling data is available in the community facilitating the curation of more training data, machine learning can significantly improve DRAGEN accuracy.

Genetalks pipeline (Participant ID: XEMVH)

We submitted three pipelines for this challenge, the pipeline diagrams were illustrated in Supplemental Figure 4.


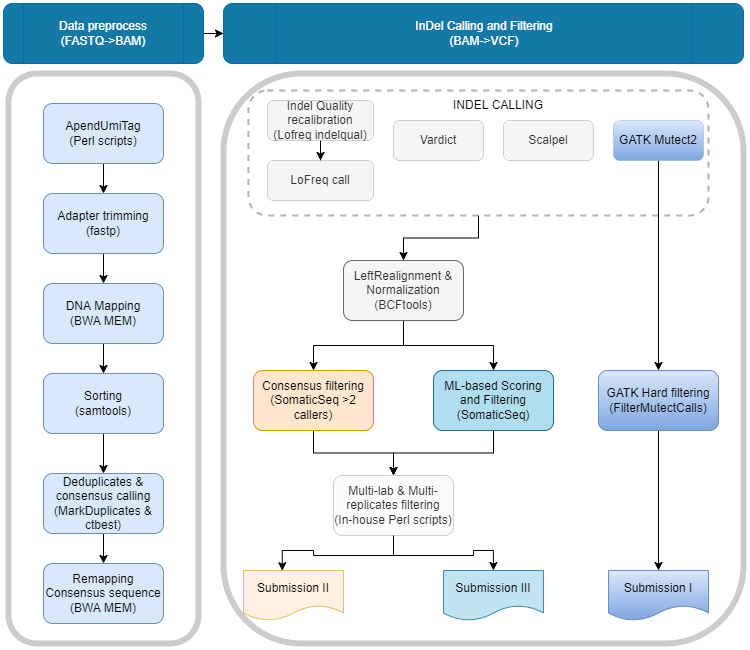


**Supplemental Figure 4**: Workflow diagrams for NCTR INDELs challenge from Genetalks®

The three pipelines shared common steps in quality control and reads mapping and distinct from each other in INDELs calling. In submission one, we use best practice recommendations in GATK Mutect2. In the other two submissions, we adopted an ensemble-based approach to merge multiple callsets using SomaticSeq pipelines. To filter false positive INDELs and maximize the overall performance, we trained a classifier for tumor-only data based on BAMsurgeon and SomaticSeq. Among all submissions, SomaticSeq-ML(submission 3) got the best performance on Oncopanel A (see Supplemental Figure 5).

**Supplemental Figure 5**: Performance of three pipelines on Oncopanel A

We believe that machine learning is a promising approach to distinguish true variants from technique artifacts. Though we simulated different length distributions and different VAF distributions to maximize the coverage of real cases in generating simulation data, according to previous studies, using simulated data combined with real data to train classifiers can achieve higher accuracy than using only simulated data. In the selection of real data, we can actually select the benchmark data of SEQC2 tumor-normal paired samples, but this part of data contains less information about challenging indels sites, which is a common case in this challenge. In addition, due to the differences in sequencing depth and VAF, the performance of the model is not ideal. Therefore, using part of the match data to train the model is also a last resort. We believe this issue will improve dramatically as more benchmark data is produced.

Here we describe the details about methods and tools used in SomaticSeq-ML.

*Read processing and mapping*

Raw reads were preprocessed by fastp(0.23.2)^9^ with parameters “--disable_quality_filtering --detect_adapter_for_pe”. The processed sequence data were mapped to the human reference genome GRCh37/hg19 using BWA(0.7.17)^10^ with parameters “-M -Y -C -K 1000000000”.

*Mark duplicated reads*

For PanelA, duplicated reads were detected and consensus sequence were called using ctbest^11^, an in-house software to process cfDNA/ctDNA NGS sequencing data. The consensus sequences were remapped to GRCh37/hg19 using BWA.

*Local realignment and BQSR*

No local alignment optimization was performed. No BQSR was performed by GATK(4.2.6.1)^6^.

*Variants calling*

We used four callers to detect INDELs and ensembled the four callsets using SomaticSeq(v3.7.3)^12^.The version of four callers and the parameters used were listed as follows:

(1) GATK Mutect2(4.2.6.1): “--max-reads-per-alignment-start 0 --initial-tumor-lod 1.0 --tumor-lod-to-emit 1.0 --max-num-haplotypes-in-population 512 --minimum-mapping-quality 10”;

(2) VarDict(1.8.3)^13^: The threshold of allele fraction(option “-f”) were set to 0.01, 0.003 for panelA and PanelB respectively together with additional parameters “-c 1 -S 2 -E 3 -g 4 -U ”;

(3) LoFreq(v2.1.5-17-g007a8ba)^14^: The BAM file was firstly processed to add “BI/BD” INDELs quality tags using function “INDELsqual”. INDELs were called for each library using “lofreq call” with parameters “-m 10 --call-INDELs --only-INDELs --use-orphan --no-default-filter”;

(4) Scalpel(0.5.4)^15^: We ran “scalpel-discovery –single” with default parameters.

*Make the training data*

To make a INDELs-free BAM, an in-house Perl script was used to remove “I/D” from BAM CIAGR and the sequences were modified to match reference sequence. The modified INDELs-free BAM files were used to simulate spike-in INDELs using modified BamSimulator in SomaticSeq package. Various options was used to control the distribution of allelic fraction and INDELs length. About >10000 spike-in INDELs were simulated.

Also, we analyzed the reproducibility of INDELs call between different labs and libraries using bcftools(1.12)^16^ using function “bcftools isec”. INDELs detected in less than 3 libraries were labeled as false positives and added to the train set.

All features were extracted using modified SomaticSeq. We modified SomaticSeq mainly because it can't extract correct `DP4` information for insertions at multiple allelic sites.

*Model training*

We train a classifier using “somatic_xgboost.py” in SomaticSeq package with parameters “-threads 72 -depth 16 -seed 579 -iter 1000 --extra-params scale_pos_weight:0.1 grow_policy: lossguide max_leaves:16”. The result model showed a high specificity and nearly filtered all multiple allelic INDELs, which was a common case in this challenge. We manually inspected some multiple allelic INDELs and added a carefully-curated list of sites to the original training set. We retrained the model using “somatic_xgboost.py” based on the new input. The input were split into training set (18688, 80%) and validation set(4673, 20%)(see Supplemental Figure 6). The validation set was used to stop the training process early to avoid overfitting. The training process stopped after 93 iterations and the result model was saved.


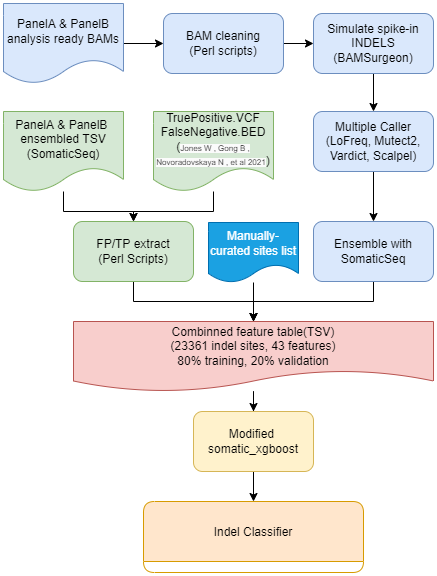


**Supplemental Figure 6**: Training data source

*Model apply and further filtering for multiple allelic sites*

The above model was applied to callsets from all libraries and INDELs classified as “PASS” were retained to be candidates. Callsets from all libraries were merged, and for multiple allelic sites, we retained only one insertion and one deletion with the highest mean QUAL score. Other alleles were recorded in a blocklist named “Panel[AB].blocklist.tsv”. INDELs passed in less than 6 libraries were also recorded in the blocklist.

Then, we used the following criteria to filter the ensembled callset:

1. deletions located crossing region borders were removed;
2. INDELs located in negative region were also removed. The negative BED were downloaded from <https://figshare>.com/articles/dataset/Consensus_Target_Region/13511829/3.
3. INDELs longer than 50nt were removed;
4. INDELs recorded in blocklist were filtered out.

Konstantinos Kyriakidis (Participant: R6CG9)

The most important factor in every variant calling pipeline and especially in a somatic variant calling pipeline is the mapping. An incorrect mapping will result in increased spurious FP variants, especially in low complexity regions. All variant calling software relies on the provided mappings. In somatic variant calling, where we can have low frequency variants due to clonality, we need to map as accurately as possible. For Illumina data, we believe the most accurate mapper right now is the DRAGEN mapper. However, due to not having a license to run it, we used the open-source version of it, DRAGMAP, which is inferior in performance.

UMIs could potentially help with variant calling, however, during preprocessing we saw that the data were already processed somehow. Therefore, we could not use the UMI information. Most UMI barcodes had 1-3 counts in the whole dataset.

One of the downsides of this competition was that we needed to report our results with respect to the hg19 reference genome. We understand that all the previous efforts to generate this benchmark dataset relied on hg19. However, mapping in this reference genome can yield a high number of FPs. We suspect that mapping to a newer reference genome such as the T2Tv2 reference genome of a Pan-Genome reference genome would result in better results, especially in difficult to map areas and low complexity regions.

Most of the currently available tumor-only variant callers generate a very high number of FPs. So, we needed to combine information from the best of them and rely on clever filtering to reduce FPs while maintaining high Recall. Mutect2 is one of the best tumor-only variant callers. The final set of variants it generates that PASS all filters are really high-quality variants that one can trust. The problem is that it generates a lot of variants that tag them as “slippage” that are true variants. All of these “slippage” variants are in homopolymer areas.

To rescue most of the true variants, we relied on another variant caller named UVC, which is based on Mutect2 and calls small variants using universality with Bayes-factor-adjusted odds ratios. We have found that it generates very good quality scores for each variant it calls and can rely on this quality score to further filter the spurious “Slippage” variants I detect with Mutect2, so we can retrieve as many TP variants as possible.

The most interesting thing we noticed working in a lot of projects with tumor data is that we found a pattern in those “Slippage” tagged variants from Mutect2 that helped filter efficiently these variants and retrieve a lot of TPs. Certain combinations of variables like STRLEN (Total STR length) and RC (the length of the sequence that is repeated in the STR segment) seem to be TPs most of the time. The filtering approach is quite complex to write here, in a few sentences, but you can check it out in the Summary file.

We believe that we have reached the peak regarding the information we can recover from short reads and no further improvements in software modeling can help us reach new frontiers in somatic variant calling. Long read sequencing is the future in this area. The length of those reads as well as the methylation patterns in those reads can help us effectively phase variants and detect and filter low frequency FPs ones.

We will focus on improving our pipeline using Long read sequencing data, especially Nanopore data. The combination of long reads and methylation will lead to new undiscovered insights on cancer progression and will become the main method for variant calling, especially for somatic variant calling.

*Read processing*

Raw reads were adapter trimmed using BBTools' bbduk (v38.96)^17^ with parameters "ref=~/bbmap/resources/adapters.fa ktrim=r k=23 mink=11 hdist=1 tpe tbo".

*Map to the reference genome*

The processed sequence data were mapped to the human reference genome GRCh37/hg19 using Illumina's DRAGMAP (v1.3.0)^18^ with the additional parameters "--preserve-map-align-order true, --RGID SampleName and --RGSM SampleName".

*Remove deduplicated reads*

Duplicate reads were detected and marked (not removed) using GATK's MarkDuplicatesSpark (v4.2.6.1)^6^ using the default parameters.

*Base Quality Score Recalibration*

Base Quality Score Recalibration (BQSR) was performed using GATK's BaseRecalibratorSpark (v4.2.6.1) using the dbSNP file (v154)^19^ in the "--known-sites" parameter.

*Variant calling*

Variant calling was performed with GATK's Mutect2 (v4.2.6.1) and Vardict (v1.8.2)^13^ through the bcbio-nextgen analysis platform (v1.2.9)^20^, using the "min_allele_fraction: 0.9" parameter in bcbio-nextgen (sites with AF less than 0.009 get a MinAF tag in FILTER column) and avoiding calling in low complexity regions using the "remove_lcr: true" parameter in bcbio-nextgen. Bcbio uses a Low Complexity Region (LCR) bed file provided in the Heng Li's variant artifacts paper^21^. Variant calling with bcbio restricted to the defined regions provided by PrecisionFDA in BED files. The low complexity regions BED file was downloaded and processed using the following command:

"wget --no-check-certificate -O - https://github.com/lh3/varcmp/raw/master/scripts/LCR-hs37d5.bed.gz | gunzip -c | grep -v ^GL | grep -v ^NC | grep -v ^hs | sed 's/^/chr/' | bgzip -c > LCR.bed.gz".

Variant calling was also performed using UVC (v0.12.01)^22^ with the additional parameters "--outvar-flag 0x4 and --sample SampleName". No Blocklist file was used with UVC. Variant calling with UVC was restricted to the defined regions provided by PrecisionFDA in BED files.

*Post processing*

The reported VCF files were further processed with a custom in-house bash script to obtain the final filtered indels. The following tools where used: GATK (v4.2.6.1), vembrane (v0.8.0)^6^, bcftools (v1.15.1)^16^ and bedtools (v2.30.0)^23^. Identity information was manually removed and filedate format was manually reformatted according to the instructions provided by the PrecisionFDA. The following post processing steps were performed:

1. UVC indels with "PASS" or "Q60" FILTER tags were extracted in a separate file using vembrane filter function.
2. UVC indels with "Q50" FILTER tag were extracted in a separate file using vembrane filter function.
3. UVC indels with "Q40" or "Q30" or "Q20" or "Q10" FILTER tags were extracted in a separate file using vembrane filter function.
4. Mutect2 indels were left aligned, trimmed and multiallelic variants were decomposed using GATK's LeftAlignAndTrimVariants function.
5. Mutect2 indels with 'FILTER~"MinAF" || FILTER~"map_qual" || FILTER~"base_qual" || FILTER~"strand_bias" || FILTER~"weak_evidence" || FILTER~"strict_strand" || FILTER~"possible_numt" || FILTER~"position" || FILTER~"orientation" || FILTER~"FAIL" | FILTER~"duplicate" || FILTER~"n_ratio" || FILTER~"low_allele_frac" || FILTER~"fragment" || FILTER~"contamination" || FILTER~"normal_artifact" were *excluded* from file (4) using bcftools view function.
6. Mutect2 indels with '(FILTER="PASS" and INFO/TLOD > 10 && INFO/STR=0) or (FILTER="PASS" && INFO/TLOD > 10 && INFO/STR=1 && INFO/STRQ >= 20)' were extracted in a separate file using bcftools view function.
7. Common indels found in both (3) and (6) files were extracted in a new file using bcftools view function.
8. Mutect2 indels with '(FILTER!~"slippage" && FILTER!="PASS" && INFO/TLOD > 20 && INFO/STR=0) || (FILTER!~"slippage" && FILTER!="PASS" && INFO/TLOD > 20 && INFO/STR=1 && INFO/STRQ >= 20)' were extracted in a new file using bcftools view function.
9. Common indels found in both (3) and (8) files were extracted in a new file using bcftools view function.
10. Mutect2 indels with 'FILTER~"slippage" && INFO/TLOD > 10' were extracted in a new file using bcftools view function.
11. Common indels found in both (3) and (10) files were extracted in a new file using bcftools view function.
12. Indels in (8) with '(STRLEN(RU)*RC <= 6 && (STRLEN(REF)-STRLEN(ALT) >= 6 || STRLEN(ALT)-STRLEN(REF) >= 6) && (INFO/tAD[1]/(INFO/tAD[1]+INFO/tAD[0]) >= 0.01) && INFO/tAD[1] > 1)' were extracted in a new file using bcftools view function.
13. Indels in (10) with '(STRLEN(RU) == 1 && RC <= 7 && (STRLEN(REF)-STRLEN(ALT) >= 8 || STRLEN(ALT)-STRLEN(REF) >= 8) && (INFO/tAD[1]/(INFO/tAD[1]+INFO/tAD[0]) >= 0.01) && INFO/tAD[1] > 1) || (STRLEN(RU) == 2 && RC <= 3 && (STRLEN(REF)-STRLEN(ALT) >= 8 || STRLEN(ALT)-STRLEN(REF) >= 8) && (INFO/tAD[1]/(INFO/tAD[1]+INFO/tAD[0]) >= 0.01) && INFO/tAD[1] > 1) || (STRLEN(RU) == 3 && (RC == 1 || RC == 2) && (STRLEN(REF)-STRLEN(ALT) >= 8 || STRLEN(ALT)-STRLEN(REF) >= 8) && (INFO/tAD[1]/(INFO/tAD[1]+INFO/tAD[0]) >= 0.01) && INFO/tAD[1] > 1) || (STRLEN(RU) == 3 && RC == 3 && (STRLEN(REF)-STRLEN(ALT) >= 3 || STRLEN(ALT)-STRLEN(REF) >= 3) && (INFO/tAD[1]/(INFO/tAD[1]+INFO/tAD[0]) >= 0.01) && INFO/tAD[1] > 1) || (STRLEN(RU) == 3 && RC == 4 && (STRLEN(REF)-STRLEN(ALT) >= 3 || STRLEN(ALT)-STRLEN(REF) >= 3) && (INFO/tAD[1]/(INFO/tAD[1]+INFO/tAD[0]) >= 0.02) && INFO/tAD[1] > 1) || (STRLEN(RU) == 4 && RC == 1 && (STRLEN(REF)-STRLEN(ALT) >= 8 || STRLEN(ALT)-STRLEN(REF) >= 8) && (INFO/tAD[1]/(INFO/tAD[1]+INFO/tAD[0]) >= 0.01) && INFO/tAD[1] > 1) || (STRLEN(RU) == 5 && RC == 1 && (STRLEN(REF)-STRLEN(ALT) >= 8 || STRLEN(ALT)-STRLEN(REF) >= 8) && (INFO/tAD[1]/(INFO/tAD[1]+INFO/tAD[0]) >= 0.01) && INFO/tAD[1] > 1) || (STRLEN(RU) == 6 && RC == 1 && (STRLEN(REF)-STRLEN(ALT) >= 8 || STRLEN(ALT)-STRLEN(REF) >= 8) && (INFO/tAD[1]/(INFO/tAD[1]+INFO/tAD[0]) >= 0.01) && INFO/tAD[1] > 1)' were extracted in a new file using bcftools view function.
14. (7), (12) and (13) files were merged into new files using the gatk MergeVcfs function.
15. Common indels found in both (2) and (6) files were extracted in a new file using bcftools view function.
16. Common indels found in both (2) and (8) files were extracted in a new file using bcftools view function.
17. Common indels found in both (2) and (10) files were extracted in a new file using bcftools view function.
18. 18. Indels in (17) with '(STRLEN(RU) == 1 && RC <= 7 && (STRLEN(REF)-STRLEN(ALT) >= 8 || STRLEN(ALT)-STRLEN(REF) >= 8) && (INFO/tAD[1]/(INFO/tAD[1]+INFO/tAD[0]) >= 0.01) && INFO/tAD[1] > 1) || (STRLEN(RU) == 2 && RC <= 3 && (STRLEN(REF)-STRLEN(ALT) >= 8 || STRLEN(ALT)-STRLEN(REF) >= 8) && (INFO/tAD[1]/(INFO/tAD[1]+INFO/tAD[0]) >= 0.01) && INFO/tAD[1] > 1) || (STRLEN(RU) == 2 && RC == 4 && (STRLEN(REF)-STRLEN(ALT) >= 2 || STRLEN(ALT)-STRLEN(REF) >= 2) && (INFO/tAD[1]/(INFO/tAD[1]+INFO/tAD[0]) >= 0.01) && INFO/tAD[1] > 1) || (STRLEN(RU) == 2 && RC == 5 && (STRLEN(REF)-STRLEN(ALT) >= 4 || STRLEN(ALT)-STRLEN(REF) >= 4) && (INFO/tAD[1]/(INFO/tAD[1]+INFO/tAD[0]) >= 0.01) && INFO/tAD[1] > 1) || (STRLEN(RU) == 2 && (RC == 6 || RC == 7 || RC == 8) && (STRLEN(REF)-STRLEN(ALT) >= 6 || STRLEN(ALT)-STRLEN(REF) >= 6) && (INFO/tAD[1]/(INFO/tAD[1]+INFO/tAD[0]) >= 0.01) && INFO/tAD[1] > 1) || (STRLEN(RU) == 3 && (RC == 1 || RC == 2) && (STRLEN(REF)-STRLEN(ALT) >= 8 || STRLEN(ALT)-STRLEN(REF) >= 8) && (INFO/tAD[1]/(INFO/tAD[1]+INFO/tAD[0]) >= 0.01) && INFO/tAD[1] > 1) || (STRLEN(RU) == 3 && RC == 3 && (STRLEN(REF)-STRLEN(ALT) >= 3 || STRLEN(ALT)-STRLEN(REF) >= 3) && (INFO/tAD[1]/(INFO/tAD[1]+INFO/tAD[0]) >= 0.01) && INFO/tAD[1] > 1) || (STRLEN(RU) == 3 && RC == 4 && (STRLEN(REF)-STRLEN(ALT) >= 3 || STRLEN(ALT)-STRLEN(REF) >= 3) && (INFO/tAD[1]/(INFO/tAD[1]+INFO/tAD[0]) >= 0.01) && INFO/tAD[1] > 1) || (STRLEN(RU) == 3 && RC == 5 && (STRLEN(REF)-STRLEN(ALT) >= 6 || STRLEN(ALT)-STRLEN(REF) >= 6) && (INFO/tAD[1]/(INFO/tAD[1]+INFO/tAD[0]) >= 0.01) && INFO/tAD[1] > 1) || (STRLEN(RU) == 3 && (RC == 6 || RC == 7 || RC == 8) && (STRLEN(REF)-STRLEN(ALT) >= 9 || STRLEN(ALT)-STRLEN(REF) >= 9) && (INFO/tAD[1]/(INFO/tAD[1]+INFO/tAD[0]) >= 0.01) && INFO/tAD[1] > 1) || (STRLEN(RU) == 4 && RC <= 4 && (STRLEN(REF)-STRLEN(ALT) >= 4 || STRLEN(ALT)-STRLEN(REF) >= 4) && (INFO/tAD[1]/(INFO/tAD[1]+INFO/tAD[0]) >= 0.01) && INFO/tAD[1] > 1) || (STRLEN(RU) == 4 && RC == 5 && (STRLEN(REF)-STRLEN(ALT) >= 8 || STRLEN(ALT)-STRLEN(REF) >= 8) && (INFO/tAD[1]/(INFO/tAD[1]+INFO/tAD[0]) >= 0.01) && INFO/tAD[1] > 1) || (STRLEN(RU) == 4 && RC >= 6 && (STRLEN(REF)-STRLEN(ALT) >= 12 || STRLEN(ALT)-STRLEN(REF) >= 12) && (INFO/tAD[1]/(INFO/tAD[1]+INFO/tAD[0]) >= 0.01) && INFO/tAD[1] > 1) || (STRLEN(RU) == 5 && RC <= 4 && (STRLEN(REF)-STRLEN(ALT) >= 5 || STRLEN(ALT)-STRLEN(REF) >= 5) && (INFO/tAD[1]/(INFO/tAD[1]+INFO/tAD[0]) >= 0.01) && INFO/tAD[1] > 1) || (STRLEN(RU) == 5 && RC == 5 && (STRLEN(REF)-STRLEN(ALT) >= 10 || STRLEN(ALT)-STRLEN(REF) >= 10) && (INFO/tAD[1]/(INFO/tAD[1]+INFO/tAD[0]) >= 0.01) && INFO/tAD[1] > 1) || (STRLEN(RU) == 5 && RC >= 6 && (STRLEN(REF)-STRLEN(ALT) >= 15 || STRLEN(ALT)-STRLEN(REF) >= 15) && (INFO/tAD[1]/(INFO/tAD[1]+INFO/tAD[0]) >= 0.01) && INFO/tAD[1] > 1) || (STRLEN(RU) == 6 && RC <= 4 && (STRLEN(REF)-STRLEN(ALT) >= 6 || STRLEN(ALT)-STRLEN(REF) >= 6) && (INFO/tAD[1]/(INFO/tAD[1]+INFO/tAD[0]) >= 0.01) && INFO/tAD[1] > 1) || (STRLEN(RU) == 6 && RC == 5 && (STRLEN(REF)-STRLEN(ALT) >= 12 || STRLEN(ALT)-STRLEN(REF) >= 12) && (INFO/tAD[1]/(INFO/tAD[1]+INFO/tAD[0]) >= 0.01) && INFO/tAD[1] > 1) || (STRLEN(RU) == 6 && (RC == 6 || RC == 7 || RC == 8) && (STRLEN(REF)-STRLEN(ALT) >= 18 || STRLEN(ALT)-STRLEN(REF) >= 18) && (INFO/tAD[1]/(INFO/tAD[1]+INFO/tAD[0]) >= 0.01) && INFO/tAD[1] > 1)' were extracted in a new file using bcftools view function.
19. Extract indels in (2) not found in (5), keeping only indels with '(STRLEN(REF)-STRLEN(ALT) >= 6 || STRLEN(ALT)-STRLEN(REF) >= 6) && INFO/tAD[1] > 1'.
20. Merge (14), (15), (16), (18), (1) and (19) vcf files.
21. Annotate the (20) VCF file according to the instructions provided by the PrecisionFDA (adding INFO/AF, INFO/DP, INFO/ADP) using awk, cut, bcftools query and bcftools annotate functions.
22. Manually remove Identity information and manually reformat filedate format according to the instructions provided by the PrecisionFDA.

Team ObiWan (Participant ID: AWJ0W)

The pipeline adopts a conventional strategy of voting, with no use of AI. Filtering and voting criteria are implemented in ad-hoc scripts. Genotyping based on DNA sequencing methods is still a challenging task. There are several software tools available (being GATK toolkit adopted in best practice protocols). An accurate and precise list of variants depends on the methods employed in each step of the genotyping:

1. No pipeline will generate an accurate and precise VCF if the DNA sequencing equipment does not read the base correctly.
2. No pipeline will generate an accurate and precise VCF if the base calling algorithm does not convert the base read signal in the correct digital base (A, C, T, G – in, e.g., FASTA/FASTQ format) or does not correctly estimate the quality statistic of the call.
3. No pipeline will generate an accurate and precise VCF if the reads in FASTA/FASTQ format are not correctly aligned against a reference sequence (considering that this reference is itself incomplete or inaccurate).
4. No pipeline will generate an accurate and precise VCF if the list of variants is not correctly identified from the alignment (e.g., BAM format).

The NCTR Indel Calling from Oncopanel Sequencing Challenge addressed (3) and (4) above. With the current technology, the point was a challenge inside a challenge: to solve the homopolymers/low complexity regions from reads whose bases were already called by the base caller. Identification of SNPs and Indels in high complexity regions (and no repeated genomic regions) is well established. However, the context of the challenge is oncogenetics and an additional task is to separate low frequency variants from noise (e.g., artifacts). Given these considerations, high accuracy and precision were achieved:

- By genotypers adapted to low frequency variant detection.
- By the voting strategy itself.
- By a meaningful set of genotyper parameters.
- By the chosen rule of filtering.

The proposed pipeline was not applied to panel A and X and no evidence exist related to its performance (if consistent/repeatable or not).

One alternative pipeline included a step of checking frequency of identified variants (mainly indels). There are databases that provide additional information related to quality of the variant based on AI genotyping algorithms. Curiously, this check did not improve the performance of the pipeline. One possible hint to explain this behavior is that AI techniques are not yet consolidated and there is still risk of validating as real an artifact, using the results generated by third party.

The pipeline proposed here had in mind a very pragmatic and simple approach. When explaining the pipeline to the geneticists, they support voting approaches. Voting does not exclude AI methods. However, the success of AI methods depends on an adequate training data and testing model. Given the experience of other alternative pipeline considering genotyping scores based on AI methods, it seems that these methods still have space for improvement.

Besides populational frequency, do not trust public databases with variant quality info. At least for indels in low complexity regions, homopolymers or variants in repeated genomic regions. Voting is still an efficient and practical approach (the two best results for panel B used voting as decision protocol). However, AI techniques and tools are improving fast, and we believe that these methods will surpass those based on voting (or non-voting) of non-IA based tools. One evidence of voting approaches is that, for panel B, this strategy was better than approaches adopted by much more sophisticated tools (e.g., Illumina Dragen).

One important improvement, if we could extend the scope of the challenge, was to improve base calling, based on AI/ML approaches. This strategy has been very successful for long read sequencing. However, for short read sequencing, in a practical framework, pipelines trust the default base caller. However, mainly to solve homopolymer (and low complexity regions) genotyping, it would be interesting to test existent tools or develop a new one in order to polish/correct the base called reads.

Summary of DeepGraph algorithm (Participant ID: HKK8M)

For this submission, we processed the raw reads by fastp^9^ version 0.23.2 with parameters "--trim_tail1 1". Then, we mapped the sequencing data to graph human reference genome hg19 using HISAT2^24^ version 2.2.1 with “--no-spliced-alignment” parameters. The graph hg19 was built by HISAT2 using UCSC hg19 reference genome with 1000 genomes^25^ variants at minimum 20% allele frequency, COSMIC^26^ v94 variants observed in greater than 2 samples and SEQC2 high confidence calls for 10 cell line mixture^1^.

Then we used fgbio^27^ AnnotateBamWithUmis to add UMI tags to the BAM file when available. If UMI tags were not available, “RX:Z:AAAAAAAA” was added. Deduplicated reads were detected by fgbio version 2.0.1 using function GroupReadsByUmi with parameters "-s Identity -e 0". Then a deep learning-based consensus sequence algorithm was used to generate consensus reads for each group of duplicate reads.

To call variants from the consensus reads, we then used a deep learning based variant caller along with other callers like MuTect2^28^, Strelka2^7^, and VarDict^13^. Octopus^29^ was also used to refine/add-to the calls and post-process the indels.

References

1 Deveson, I. W. *et al.* Evaluating the analytical validity of circulating tumor DNA sequencing assays for precision oncology. *Nat Biotechnol* **39**, 1115-1128, doi:10.1038/s41587-021-00857-z (2021).

2 Golkaram, M. *et al.* *DRAGEN High Accuracy Indel Calling Wins PrecisionFDA NCTR Indel Calling from Oncopanel Sequencing Data Challenge*, <<https://www.illumina.com/science/genomics-research/articles/precisionfda-indel.html>> (2022).

3 Olson, N. D. *et al.* PrecisionFDA Truth Challenge V2: Calling variants from short and long reads in difficult-to-map regions. *Cell Genom* **2**, doi:10.1016/j.xgen.2022.100129 (2022).

4 Mehio, R. *et al.* *DRAGEN Wins at PrecisionFDA Truth Challenge V2 Showcase Accuracy Gains from Alt-aware Mapping and Graph Reference Genomes*, <<https://www.illumina.com/science/genomics-research/articles/dragen-wins-precisionfda-challenge-accuracy-gains.html>> (2020).

5 Betschart, R. O. *et al.* Comparison of calling pipelines for whole genome sequencing: an empirical study demonstrating the importance of mapping and alignment. *Sci Rep* **12**, 21502, doi:10.1038/s41598-022-26181-3 (2022).

6 Van der Auwera, G. A. & O'Connor, B. D. *Genomics in the Cloud*. 1st edn, (O'Reilly Media, Inc., 2020).

7 Kim, S. *et al.* Strelka2: fast and accurate calling of germline and somatic variants. *Nat Methods* **15**, 591-594, doi:10.1038/s41592-018-0051-x (2018).

8 Scheffler, K. *et al.* Accuracy improvements in somatic whole-genome small-variant calling with the DRAGEN platform. *Cancer Research* **80**, 5463, doi:10.1158/1538-7445.AM2020-5463 (2020).

9 Chen, S., Zhou, Y., Chen, Y. & Gu, J. fastp: an ultra-fast all-in-one FASTQ preprocessor. *Bioinformatics* **34**, i884-i890, doi:10.1093/bioinformatics/bty560 (2018).

10 Li, H. Aligning sequence reads, clone sequences and assembly contigs with BWA-MEM. arXiv:1303.3997 (2013). <<https://ui.adsabs.harvard.edu/abs/2013arXiv1303.3997L>>.

11 Yang, X. *et al.* A Cell-free DNA Barcode-Enabled Single-Molecule Test for Noninvasive Prenatal Diagnosis of Monogenic Disorders: Application to beta-Thalassemia. *Adv Sci (Weinh)* **6**, 1802332, doi:10.1002/advs.201802332 (2019).

12 Fang, L. T. in *Bioinformatics for Cancer Immunotherapy: Methods and Protocols* (ed Sebastian Boegel) 47-70 (Springer US, 2020).

13 Lai, Z. *et al.* VarDict: a novel and versatile variant caller for next-generation sequencing in cancer research. *Nucleic Acids Res* **44**, e108, doi:10.1093/nar/gkw227 (2016).

14 Wilm, A. *et al.* LoFreq: a sequence-quality aware, ultra-sensitive variant caller for uncovering cell-population heterogeneity from high-throughput sequencing datasets. *Nucleic Acids Res* **40**, 11189-11201, doi:10.1093/nar/gks918 (2012).

15 Fang, H. *et al.* Indel variant analysis of short-read sequencing data with Scalpel. *Nat Protoc* **11**, 2529-2548, doi:10.1038/nprot.2016.150 (2016).

16 Danecek, P. *et al.* Twelve years of SAMtools and BCFtools. *Gigascience* **10**, doi:10.1093/gigascience/giab008 (2021).

17 Bushnell, B. *BBTools*, <<https://jgi.doe.gov/data-and-tools/software-tools/bbtools/>> (

18 Rizk, G. *DRAGMAP*, <<https://github.com/Illumina/DRAGMAP>> (2022).

19 Sherry, S. T., Ward, M. & Sirotkin, K. dbSNP-database for single nucleotide polymorphisms and other classes of minor genetic variation. *Genome Res* **9**, 677-679 (1999).

20 Chapman, B. *et al.* bcbio/bcbio-nextgen: (v1.2.9). *Zenodo* **2023** (2021). <<https://doi.org/10.5281/zenodo.5781867>>.

21 Li, H. Toward better understanding of artifacts in variant calling from high-coverage samples. *Bioinformatics* **30**, 2843-2851, doi:10.1093/bioinformatics/btu356 (2014).

22 Zhao, X., Hu, A. C., Wang, S. & Wang, X. Calling small variants using universality with Bayes-factor-adjusted odds ratios. *Briefings in Bioinformatics* **23**, doi:10.1093/bib/bbab458 (2021).

23 *bedtools - the swiss army knife for genome arithmetic*, <<https://github.com/arq5x/bedtools2>> (2021).

24 Kim, D., Paggi, J. M., Park, C., Bennett, C. & Salzberg, S. L. Graph-based genome alignment and genotyping with HISAT2 and HISAT-genotype. *Nat Biotechnol* **37**, 907-915, doi:10.1038/s41587-019-0201-4 (2019).

25 Genomes Project, C. *et al.* A global reference for human genetic variation. *Nature* **526**, 68-74, doi:10.1038/nature15393 (2015).

26 Tate, J. G. *et al.* COSMIC: the Catalogue Of Somatic Mutations In Cancer. *Nucleic Acids Res* **47**, D941-D947, doi:10.1093/nar/gky1015 (2019).

27 *fgbio*, <<https://github.com/fulcrumgenomics/fgbio>> (2022).

28 Cibulskis, K. *et al.* Sensitive detection of somatic point mutations in impure and heterogeneous cancer samples. *Nat Biotechnol* **31**, 213-219, doi:10.1038/nbt.2514 (2013).

29 Cooke, D. P., Wedge, D. C. & Lunter, G. A unified haplotype-based method for accurate and comprehensive variant calling. *Nat Biotechnol* **39**, 885-892, doi:10.1038/s41587-021-00861-3 (2021).
